# Quantitative phosphoproteomic analyses identify STK11IP as a lysosome-specific substrate of mTORC1 that regulates lysosomal acidification

**DOI:** 10.1038/s41467-022-29461-8

**Published:** 2022-04-01

**Authors:** Zhenzhen Zi, Zhuzhen Zhang, Qiang Feng, Chiho Kim, Xu-Dong Wang, Philipp E. Scherer, Jinming Gao, Beth Levine, Yonghao Yu

**Affiliations:** 1 Department of Biochemistry, University of Texas Southwestern Medical Center, Dallas, TX 75390 USA; 2 Touchstone Diabetes Center, University of Texas Southwestern Medical Center, Dallas, TX 75390 USA; 3 Harold C. Simmons Comprehensive Cancer Center, University of Texas Southwestern Medical Center, Dallas, TX 75390 USA; 4 Department of Cell Biology, University of Texas Southwestern Medical Center, Dallas, TX 75390 USA; 5 Howard Hughes Medical Institute, University of Texas Southwestern Medical Center, Dallas, TX 75390 USA

**Keywords:** Nutrient signalling, TOR signalling, Lysosomes

## Abstract

The evolutionarily conserved serine/threonine kinase mTORC1 is a central regulator of cell growth and proliferation. mTORC1 is activated on the lysosome surface. However, once mTORC1 is activated, it is unclear whether mTORC1 phosphorylates local lysosomal proteins to regulate specific aspects of lysosomal biology. Through cross-reference analyses of the lysosome proteome with the mTORC1-regulated phosphoproteome, we identify STK11IP as a lysosome-specific substrate of mTORC1. mTORC1 phosphorylates STK11IP at Ser^404^. Knockout of STK11IP leads to a robust increase of autophagy flux. Dephosphorylation of STK11IP at Ser^404^ represses the role of STK11IP as an autophagy inhibitor. Mechanistically, STK11IP binds to V-ATPase, and regulates the activity of V-ATPase. Knockout of STK11IP protects mice from fasting or Methionine/Choline-Deficient Diet (MCD)-induced fatty liver. Thus, our study demonstrates that STK11IP phosphorylation represents a mechanism for mTORC1 to regulate lysosomal acidification and autophagy, and points to STK11IP as a promising therapeutic target for the amelioration of diseases with aberrant autophagy signaling.

## Abstract (web summary)

mTORC1, central regulator of cell growth and autophagy suppressor, is activated on the lysosome surface, but its local role in lysosomal biology remains unclear. Here the authors show STK11IP is a substrate of mTORC1 that regulates lysosomal acidification through V-ATPase and represses autophagy.

## Introduction

The evolutionarily conserved Ser/Thr protein kinase mTORC1 (mammalian target of rapamycin complex 1) functions as the core catalytic component of a pathway that regulates a variety of cellular anabolic processes^1,2^. mTORC1 is activated on the surface of lysosomes^2–4^. Two necessary conditions are required to activate mTORC1. First, mTORC1 is recruited to the lysosome surface by the V-ATPase-Ragulator-Rag complex in response to amino acid and other nutrients; second, mTORC1 is then activated by the lysosomal-bound GTPase, Rheb, in response to the growth factor-PI3K-AKT-TSC1/2 signaling pathway^2,5^. Recent work has focused on identifying the mechanism by which mTORC1 translocates to the lysosome in response to different nutrients^6,7^. Although the upstream modulators of mTORC1 activation have been well addressed, the downstream signaling network of mTORC1 is poorly characterized. The best-known substrates of mTORC1 are the ribosome protein S6 kinases (S6K) and the eIF4E-binding proteins (4EBPs), both of which are known to regulate protein synthesis^8^. In addition, mTORC1 regulates the synthesis of lipids and adipose tissue differentiation through SREBP and PPAR-γ^9,10^. Recently, using large-scale quantitative mass spectrometry experiments, we and others have identified additional mTORC1 substrates and targets (for example, GRB10, IGFBP5, FOXK1, and SRPK2) that are involved in a number of critical cellular anabolic processes (e.g., glycolysis and lipid synthesis)^11–16^.

A number of recent studies showed that the lysosome is a key signaling hub that communicates, in a bi-directional manner, with mTORC1. Specifically, the lysosome not only controls the activation of mTORC1, but also receives signals from mTORC1 to regulate a number of important biological processes^17^. For example, mTORC1 phosphorylates TFEB (Transcription factor EB), which is a master regulator of lysosome and autogenic gene expression. These phosphorylation events cause the binding of TFEB to the 14-3-3 proteins, and then the blockade of its translocation from the cytosol to the nucleus^17,18^. As a conserved cellular response to nutrient deprivation, mTORC1 also controls autophagy through inhibiting two key initiators of autophagosome formation, the ULK1 (Atg1) and the Atg13 proteins^19,20^.

A key question is that once mTORC1 is activated on the lysosome surface, whether mTORC1 phosphorylates, and signals through local lysosomal proteins. In this work, we integrate the lysosome immunoprecipitation (LysoIP) method^21^ with high sensitivity mass spectrometry to characterize the lysosomal proteome. Cross-reference analysis of this dataset with our previous mTORC1-related phosphoproteomic datasets^13^ points to a poorly characterized protein, STK11IP, as a potential lysosomal substrate of mTORC1. Biochemical experiments then confirmed that mTORC1 directly phosphorylates STK11IP at S404 (S404 in the human protein; S405 in the mouse or rat protein). We further show that STK11IP and its phosphorylation at S404 play a critical role in regulating lysosome acidification and autophagy. Mice with STK11IP knockout have increased autophagy levels and are protected against MCD diet- or fasting-induced nonalcoholic steatohepatitis (NASH). Mechanistically, STK11IP regulates lysosome acidification by binding to and controlling V-ATPase activity. In summary, our findings uncover a novel mechanism by which mTORC1 regulates lysosome acidification through its lysosomal-specific substrate, STK11IP. Our study also points to the exciting possibility of targeting STK11IP to therapeutically manipulate autophagy for the treatment of pathological conditions, including cancer, neurodegeneration, and age-related diseases.

## Results

### Integrative analyses of the mTORC1-regulated, lysosomal phosphoproteome

Using a SILAC (Stable isotope labeling by amino acids in cell culture)-based, quantitative phosphoproteomic approach, we previously interrogated the downstream signaling network of mTORC1, and in doing so, identified a number of novel mTORC1 substrates (e.g., Grb10)^11,12^. However, phosphoproteomic experiments at the organelle level pose a critical challenge, owing to the low stoichiometry of protein phosphorylation, and hence, the requirement for large amounts of starting materials. To overcome this issue, we developed a two-step method. In the first step, we used mass spectrometry to define the lysosome proteome, and also the global mTORC1-regulated phosphoproteome, respectively. In the second step, we cross-referenced the two datasets, and bioinformatically extracted the potential lysosome-specific mTORC1 substrates.

**Fig. 1:**
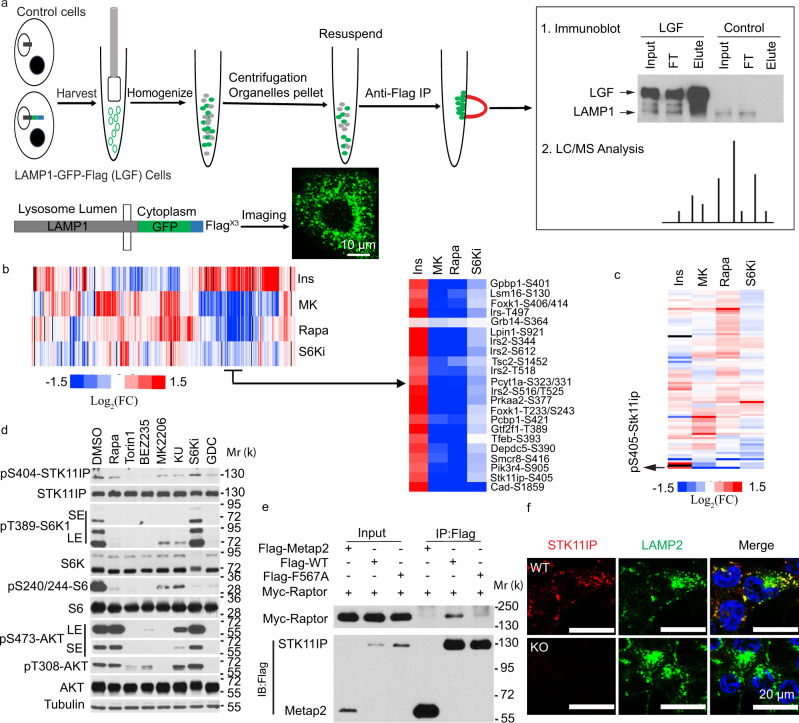
Integrative analyses of the mTORC1-regulated lysosomal phosphoproteome. **a** Schematic of the workflow for the Lysosome-IP method. HEK293T cells (control, or those stably expressing LAMP1-GFP-Flag) were harvested and homogenized. Flag-tagged lysosomes were isolated with the anti-Flag magnetic beads and subject to western blot or LC–MS/MS analyses. The confocal result showed the localization of the LAMP1-GFP signal. Scales bars, 10 μm. **b** Heatmap result of the representative phosphopeptides identified in the various hepatic quantitative phosphoproteomic datasets^13^. FC, fold change (treatment/control). The various treatment conditions can be found in ref. ^13^. In brief, Ins (the cells were treated with or without insulin); MK (the cells were treated with or without MK2206, an Akt inhibitor. Both groups were then stimulated with insulin); Rapa (the cells were treated with or without rapamycin. Both groups were then stimulated with insulin); S6Ki (the cells were treated with or without LYS6K2, an S6 kinase inhibitor. Both groups were then stimulated with insulin). **c** Heatmap representation of the phosphopeptides from proteins commonly identified in the lysosome-proteomic and hepatic phosphoproteomic datasets. *FC* fold change, pS405-Stk11ip in the rat protein (pS404 in the human protein). **d** Phosphorylation of STK11IP at S404 was blocked by mTOR inhibitors in HEK293T cells (treatment time = 4 h). DMSO treatment was used as the control; Rapamycin (Rapa, 20 nM, an mTORC1 inhibitor); Torin1 (2 μM, an mTOR kinase inhibitor); BEZ235 (1 μM, a PI3K/mTORC1 dual inhibitor); MK2206 (1 μM, an Akt inhibitor); KU-0063794 (KU, 1 μM, an mTOR kinase inhibitor); PF-4708671 (S6Ki, 10 μM, an S6K inhibitor); GDC-0941 (1 μM, a PI3K inhibitor). **e** STK11IP interacts with Raptor, and the binding is regulated by its TOS motif. F567A: the TOS motif mutant of STK11IP. **f** Immunofluorescence analysis of STK11IP (Red color) and LAMP2 (Green color, a lysosomal marker) in the wild-type and STK11IP knockout (KO) HEK293T cells. Scales bars, 20 μm.

Using a recently reported strategy^22^, we generated HEK293T cells stably expressing LAMP1 that was fused with a C-terminal GFP and Flag^3X^ tag (LGF: LAMP1-GFP-Flag^3X^) (Fig. 1a). The lysosomes were then isolated using immunoprecipitation (IP) experiments. The isolated lysosomes showed superior purity, compared to those obtained using traditional ultracentrifugation-based approaches (Supplementary Fig. 1a, b). From this lysosome-proteomic experiment (two biological replicate experiments were performed), we were able to identify a total of 231 proteins (Supplementary Dataset 1). Gene Ontology analyses showed that many of these proteins were localized in the vacuole and lysosome. These proteins were enriched with biological processes including vesical-mediated transport, endocytosis, and ion transport, all of which are known to be connected to lysosome biology (Supplementary Fig. 1c–e)^23,24^. We interrogated the list, and found that the identified proteins included both well-known lysosomal proteins, including LAMP1 and LAMP2; the Ragulator complex proteins; the V-ATPase complex proteins; hydrolases; ion transporters; trafficking and fusion machinery proteins (Supplementary Fig. 1f)^23^, as well as several recently reported lysosomal proteins, including SLC38A9^25,26^, and aldolase A^27^ (Supplementary Fig. 1f).

Using a reductive dimethylation-based quantitative mass spectrometry approach, we recently characterized the hepatic phosphoproteome (in primary rat hepatocytes) that was controlled by insulin, as well as its key downstream kinases, including Akt, mTORC1, and S6K^13^. Cross-reference analyses showed that a total of 45 proteins were commonly identified between the hepatic phosphoproteomic datasets and the lysosomal proteomic results (Fig. 1b, c and Supplementary Fig. 1g, Dataset 2). We then extracted, from the hepatic phosphoproteomic datasets, the proteins that were potential mTORC1 substrates, i.e., those proteins with decreased phosphorylation upon rapamycin treatment, and unchanged phosphorylation upon the treatment of an S6K inhibitor. Among these proteins, STK11IP was also identified in the lysosomal proteomic experiment (Fig. 1b, c).

### STK11IP is a lysosome-specific substrate of mTORC1

Phosphorylation of STK11IP (S405 in the rat/mouse protein and S404 in the human protein. The phosphorylation site in the human protein will be used hereafter) decreased markedly (approximately six fold) after rapamycin treatment (Supplementary Fig. 1h). This phosphorylation site is conserved and positioned in a sequence (i.e., LDPS^404^PAG) that is consistent with known mTORC1 phosphorylation motifs (i.e., a Pro residue at the +1 position)^28^ (Supplementary Fig. 1i). We found that phosphorylation pattern of STK11IP (pS404) was similar to other known mTORC1 substrates, ULK1 (pS757), but was different from known substrates of S6K (an AGC kinase that prefers basic residues in the vicinity of the phosphorylation site), including Cad (pS1859) and rpS6 (pS236/240) (Supplementary Fig. 1j)^20,29,30^. Collectively, these results suggested that STK11IP (pS404) could be a direct substrate of mTORC1.

We generated a phospho-specific antibody against STK11IP/pS404 (Supplementary Fig. 2a, b) and found that pS404 was potently inhibited by Rapamycin, and mTOR kinase inhibitors (e.g., Torin1 and BEZ235). This phosphorylation site, however, was refractory to inhibitors of S6K (Fig. 1d), p38 MAPK, JNK, or ERK (Supplementary Fig. 2c). pS404-STK11IP levels were also dramatically downregulated during amino acid starvation (Supplementary Fig. 2d). Moreover, mTOR was able to phosphorylate STK11IP (pS404) in an in vitro kinase assay (Supplementary Fig. 2e). We found that STK11IP interacted with Raptor, mTOR, and PRAS40, but not Rictor (Fig. 1e and Supplementary Fig. 2f–h). Finally, an inspection of the STK11IP sequence suggested that this protein contained a potential TOS motif (F^567^EVEL) (TOS motif refers to the TOR signaling motif^31^). Indeed, the mutation of the key residue (F567) in this TOS motif (F567A) abolished the binding between STK11IP and Raptor (Fig. 1e). These biochemical results further demonstrated that STK11IP was a direct substrate of mTORC1.

We then confirmed that STK11IP existed in the lysosome fraction using both IP and ultracentrifugation approaches (Supplementary Fig. 1a, b). This was further validated by inverse IP experiments using the STK11IP-GFP-Flag (SGF) stable cells, in which only lysosomes were immunoprecipitated (Supplementary Fig. 2i). Co-immunostaining assays showed that endogenous STK11IP was largely co-localized with LAMP2 (a lysosome marker that is localized on the lysosome membrane) and in part with Rab7 (an endosome, autophagosome, and lysosome marker), but not with Tom20 (a mitochondrial marker) and EEA1 (early endosome marker) (Fig. 1f and Supplementary Fig. 2j, k). Finally, we found that STK11IP was distributed in the membrane fraction using subcellular fractionation experiments (Supplementary Fig. 2l). Taken together, these results suggested that STK11IP is a mTORC1 substrate that is resident on the lysosomal membrane.

### STK11IP deficiency promotes autophagy

STK11IP is a poorly characterized protein^32,33^. A number of mTORC1 substrates are known to regulate its activation through feedback mechanisms^34,35^. However, we did not observe any differences in mTORC1 activity between the control and STK11IP knockout (KO) primary mouse embryonic fibroblasts (MEF) cells, as shown by their similar pS6K levels (Supplementary Fig. 3a). We used the CRISPR-Cas9 system to generate the STK11IP KO cells, and reconstituted these cells with WT-, S404A-, or S404D-STK11IP. We performed amino-acid starvation experiments and found no differences in mTORC1 activities in these cells (Supplementary Fig. 3b). These experiments suggest that the activation of mTORC1 is not affected by STK11IP, or its mTORC1-mediated phosphorylation.

**Fig. 2:**
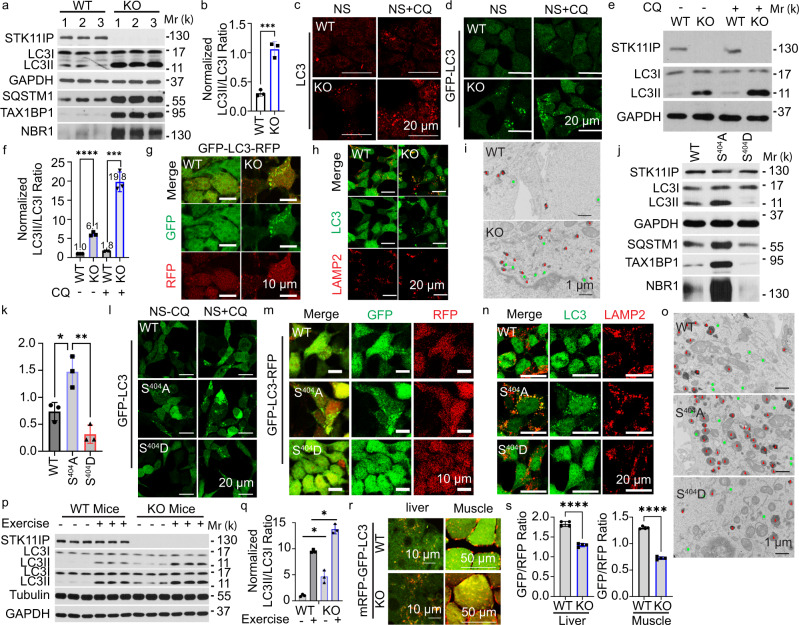
STK11IP Deletion Promotes Autophagy. **a**, **b** STK11IP depletion leads to increased autophagy. **a** Immunoblot analyses; b, quantification of the LC3II/LC3I ratio. *n* = 3 independent biology samples, ***P  =  0.0007. **c**, **d** STK11IP knockout (KO) leads to more LC3 puncta (**c** endogenous LC3; **d** GFP-LC3 puncta using the GFP-LC3 stable cells). NS (non-starvation), CQ (chloroquine, 20 μM, 2 h). Scales bars, 20 μm. **e**, **f** STK11IP knockout leads to increased LC3 turnover. *CQ* chloroquine, 20 μM, 2 h treatment. **e** Immunoblot analyses, **f** quantification of the LC3II/LC3I ratio. *n* = 3 independent biological replicates. ***P = 0.0003, ****P < 0.0001. **g** STK11IP knockout leads to increased autophagic flux. GFP (Green signals); RFP (Red signals); Scales bars, 10 μm. **h** STK11IP deletion leads to enhanced colocalization of LAMP2 (Red) and LC3 (Green). Scales bars, 20 μm. **i** STK11IP deletion leads to enhanced autolysosome formation. Double membrane autophagosomes (Green), single membrane autolysosomes (Red). Scale bars: 1 μm. **j**, **k** S404A-STK11IP mutation leads to enhanced autophagy. **j** Immunoblot analyses, **k** quantification of LC3II/LC3I ratio. *n* = 3 independent biological replicates. *P  =  0.0186; **P = 0.0038. **l** S404A-STK11IP mutation leads to the increased formation of GFP-LC3 puncta. *NS* non-starvation, *CQ* chloroquine, 20 μM, 2 h treatment. Scales bars, 20 μm. **m** S404A-STK11IP mutation leads to increased autophagic flux. GFP (Green signals); RFP (Red signals); scales bars, 10 μm. **n** S404A-STK11IP mutation leads to increased colocalization of LAMP2 (Red color) and LC3 (Green color). Scales bars, 20 μm. **o** S404A-STK11IP mutation leads to enhanced autolysosome formation. Double membrane autophagosomes (Green), single membrane autolysosomes (Red). Scale bars: 1 μm. **p**, **q** STK11IP KO leads to increased LC3II/LC3I ratios in the mice under both resting and endurance exercise conditions. **p** Immunoblot analyses; **q** quantification results of LC3II/LC3I ratio. *n* = 3 independent biology samples. *P  =  0.0426, *P  =  0.0238, respectively. **r**, **s** STK11IP KO leads to increased formation of mRFP-GFP-LC3 puncta in the liver and muscle after the endurance exercise test. **r** represent images, **s** quantification results of GFP/RFP ratio. Scales bars, 10 μm for liver, 50 μm for muscle. *n* = 4 independent biology samples. ****P  < 0.0001. All of the experiments were performed using HEK293T cells; results represent mean ± SEM; the statistical significance was calculated with unpaired two-tailed Student’s *t* tests using the GraphPad Prism software 9. Source data are provided as a Source Data file.

We next investigated whether STK11IP could mediate certain biological processes downstream of mTORC1. In particular, mTORC1 is critically involved in controlling autophagy, via several of its downstream target proteins (e.g., TFEB and ULK1)^20,23^. Here, we found that the downregulation of pS404-STK11IP occurred together with the upregulation of autophagy levels, as shown by the increased conversion of LC3I to LC3II (LC3II/LC3I ratio) upon FBS or FBS/Glucose starvation. We also observed that the downregulation of pS404-STK11IP occurred together with the degradation of LC3 upon amino acid/FBS or amino acid-only starvation conditions (Supplementary Fig. 2d). These results suggest that STK11IP may act as a downstream target of mTORC1 to regulate autophagy. To directly test this hypothesis, we knocked out STK11IP in HEK293T cells using the CRISPR-Cas9 technology. Compared to control cells, LC3II, the marker for autophagosome numbers, was markedly increased in STK11IP KO cells (Fig. 2a, b, and Supplementary Fig. 3c). Besides, there were more LC3 puncta (endogenous LC3) or GFP-LC3 puncta (using GFP-LC3 stable cells) in STK11IP KO cells compared to those in the control cells (Fig. 2c, d). STK11IP KO also leads to a profound increase in LC3 or GFP-LC3 puncta numbers under chloroquine (CQ) treatment compared to control conditions (Fig. 2c, d).

Both autophagy activation and decreased autophagic degradation will lead to the increase of the autophagosome number^36^. The LC3 turnover assay measures the autophagic flux, which is, therefore, a more reliable indicator of autophagic activity^36^. We performed the LC3 turnover assay to compare the autophagy flux between WT and STK11IP KO cells. Our results showed that STK11IP KO increases autophagy flux by around two fold (LC3II/LC3I ratio with CQ versus without CQ in WT and KO cells) (Fig. 2e, f and Supplementary Fig. 3d). Another commonly used assay for autophagic flux measurement is based on a fluorescent probe, GFP-LC3-RFP^37^. Because of the different stability between the GFP and RFP signal under acidic conditions, the GFP/RFP ratio reflects the autophagic flux^37,38^. We then applied the fluorescent probe assay and found that STK11IP KO cells displayed a lower GFP/RFP ratio (stronger yellow color) and more GFP-LC3 puncta, compared to control cells (Fig. 2g and Supplementary Fig. 3e, f), indicating the enhanced autophagy flux in the STK11IP KO cells. More specifically, we found that the formation of the autolysosomes was enhanced in STK11IP KO cells (Fig. 2h and Supplementary Fig. 3g). We observed increased colocalization of LC3 and LAMP1/LAMP2 in STK11IP−/− cells compared to wild-type cells, suggesting that there is an increased number of autolysosomes in the STK11IP−/− cells under basal conditions (Fig. 2h and Supplementary Fig. 3g). We further confirmed this using TEM (Transmission electron microscopy). The results showed that the formation of both the autophagosomes and autolysosomes (especially the autolysosomes) was highly increased in the STK11IP−/− cells compared to the wild-type cells (Fig. 2i). Thus, the autophagy flux was increased when STK11IP was deleted.

To investigate the role of the mTORC1-mediated phosphorylation of STK11IP in autophagy, we expressed the WT-, S404A-, or S404D-STK11IP in the STK11IP KO cells. Both LC3II levels and GFP-LC3 puncta numbers were increased in cells that stably expressed the S404A-STK11IP mutant, compared to WT and the S404D mutant (Fig. 2j–l). To measure the autophagy flux, we also expressed the GFP-LC3-RFP probe in the cells expressing the WT-, S404A-, or S404D-STK11IP. Similar to the STK11IP KO cells, cells expressing the S404A-STK11IP mutant showed a higher autophagy flux, as demonstrated by the reduced GFP/RFP signal and more GFP-LC3 puncta (Fig. 2m and Supplementary Fig. 3h). Furthermore, the autolysosome formation was enhanced in the S404A-STK11IP reconstituted cells compared to the WT-STK11IP or the S404D-STK11IP reconstituted cells, as shown by the increased colocalization of LC3 and LAMP2 in the S404A-STK11IP cells (Fig. 2n). This was also confirmed by the TEM results (Fig. 2o). Collectively, our results suggest that de-phosphorylation of STK11IP at S404 also promotes autophagy flux.

We next utilized the STK11IP KO mice to determine whether STK11IP regulates autophagy in vivo. One of the most potent physiological inducers of autophagy is exercise^39,40^. To test whether STK11IP can regulate exercise-induced autophagy in vivo, WT and STK11IP KO littermates were subject to the endurance exercise test (Supplementary Fig. 3i–m). We found that the STK11IP KO mice exhibited much higher levels of LC3II conversion in the soleus muscle under both resting and endurance exercise conditions, compared to the wild-type mice (Fig. 2p, q). The pS404-STK11IP level was decreased upon endurance treadmill exercise test (Supplementary Fig. 3n). We also generated a mouse line by crossing the STK11I KO mice with the TfLC3 (mRFP-GFP-LC3) mice^41^. We then assessed the number of mRFP-GFP-LC3 puncta in the liver and soleus muscle after the endurance exercise test. Remarkably, STK11IP KO mice displayed reduced GFP/RFP ratios and enhanced mRFP-GFP-LC3 puncta in both liver and soleus, which indicated a dramatic upregulation of autophagy flux in these mice (Fig. 2r, s and Supplementary Fig. 3o–q). In summary, STK11IP KO promotes autophagy flux in vivo.

### STK11IP binds to V-ATPase to regulate lysosomal acidification

We investigated the potential molecular mechanism by which STK11IP regulates autophagy. Autophagy is a multi-step process, which includes, initiation, autophagosome formation, and the autophagosome–lysosome fusion^36,42^. We did not observe any differences in the level of lysosome biogenesis and lysosome morphologies in control vs. STK11IP KO cells (Supplementary Fig. 4a, b).

**Fig. 3:**
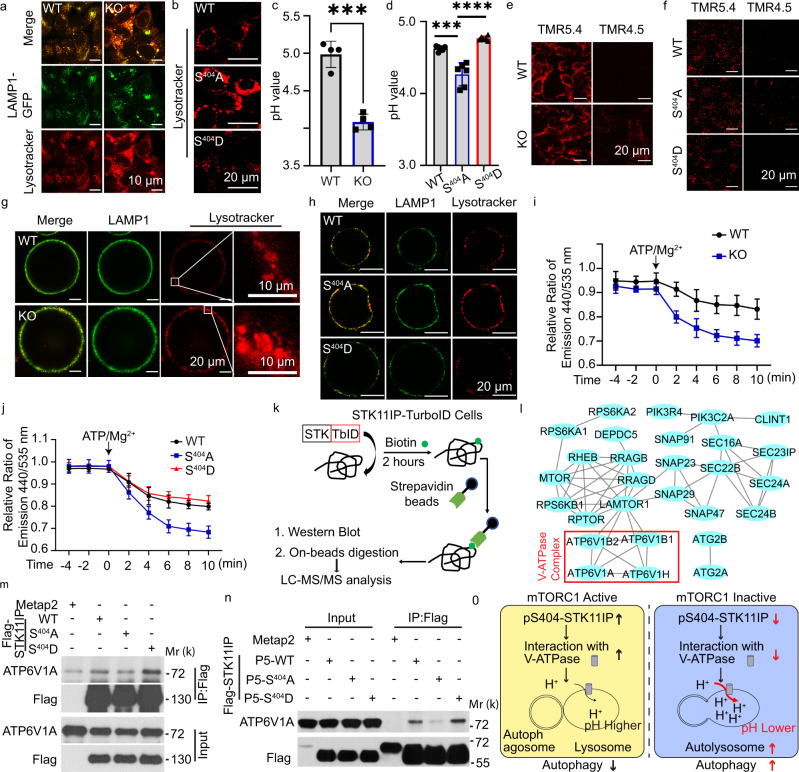
STK11IP regulates lysosomal acidification. **a** STK11IP knockout leads to increased lysosomal acidity. *WT* wild type, KO, STK11IP knockout. Scale bar, 10 μm. **b** S404A-STK11IP mutation leads to increased lysosomal acidity compared with WT/S404D-STK11IP. Scale bar, 20 μm. **c**, **d** STK11IP KO and S404A mutation lead to lower pH values compared to the control. *n* = 4 independent biology replicates. Results represent mean ± SEM. The statistical significance was calculated with unpaired two-tailed Student’s *t* tests. ***P  = 0.0001, ***P  = 0.0003, ****P  < 0.0001 respectively. **e**, **f** STK11IP KO **e** and S404A mutation **f** leads to lower pH values compared to the control using the TMR (tetramethyl rhodamine)-HyUPS nanoprobes. Scale bars, 20 μm. TMR 5.4/4.5 represent the probes with a pH threshold of 5.4 or 4.5, respectively. **g**, **h** STK11IP KO **g** and S404A-STK11IP mutation **h** leads to increased lysosomal acidity in the isolated lysosomes compared to the control cells. Scale bars = 20 μm, 10 μm for micrographs. **i** The activity of V-ATPase is enhanced in STK11IP knockout cells compared to WT cells. Where indicated, lysosomal re-acidification was initiated by the addition of ATP/Mg^2+^. (*n* = 4 independent biology samples per point), results represent mean ± SEM. **j** The activity of V-ATPase is enhanced in the S404A-STK11IP reconstituted cells compared to WT and S404D-STK11IP reconstituted HEK293T cells. The same procedure as compared to that in **i** was used to measure the V-ATPase activity. **k**, **l** Schematic of the workflow for the STK11IP-TurboID experiment (**k**). Functional association analyses of the STK11IP-interacting proteins identified from the TurboID MS experiment (**l**). The protein–protein interaction network was generated using the STRING database and was rendered using CytoScape. The V-ATPase complex components were highlighted in the red box. **m** The S404A-STK11IP mutant interacts more weakly with ATP6V1A, compared with STK11IP WT or the S404D mutant. **n** The P5 domain of STK11IP bearing the S404A mutation interacts more weakly with ATP6V1A, compared with the WT/S404D P5 mutant. P5: STK11IP (amino acid 360–770). **o** The scheme of STK11IP in regulating lysosomal acidification and autophagy. Source data are provided as a Source Data file.

As mentioned above, the GFP-LC3-RFP stable cells or the mRFP-GFP-LC3 transgenic mice showed that the GFP/RFP ratio was decreased in the STK11IP−/− cells or mice, and in the S404A-STK11IP reconstituted cells compared to the controls (Fig. 2g, m, r, s and Supplementary Fig. 3e, f, h, p, q). We hypothesized that STK11IP could regulate lysosomal acidification, because of the differential stability between the GFP and RFP proteins under acidic conditions^37,39^. mTORC1 inactivation by Torin1 treatment or amino-acid starvation could lead to the decrease of pH value (Supplementary Fig. 4c) and activation of autophagy (Supplementary Fig. 2d)^36^. These results point to the possibility that mTORC1 could regulate lysosomal acidification via a STK11IP-dependent mechanism. Indeed, we observed that the lysosomes were more acidic in STK11IP KO cells relative to the controls (Fig. 3a and Supplementary Fig. 4d). Furthermore, STK11IP KO cells reconstituted with the S404A-STK11IP mutant also showed more acidic lysosomes, compared with those derived from the cells expressing WT-STK11IP or the S404D-STK11IP mutant (Fig. 3b). We also used a ratiometric probe, LysoSensor yellow/blue dye DND-160, to measure the lysosomal pH value (based on the ratio of yellow/blue signals)^43,44^. The results showed that the pH value was significantly lower in STK11IP KO cells and S404A-STK11IP expressing cells (Fig. 3c, d and Supplementary Fig. 4e), compared to their control cells. Recently, a series of TMR (tetramethyl rhodamine)-HyUPS (hybrid ultra-pH sensitive) nanoprobes were reported, which display much sharper on/off pH responses^45,46^. We observed that the lysosomal pH value was lower than 5.4 in both the STK11IP KO cells and WT-/S404A-/S404D-STK11IP-reconstituted cells. However, the activated TMR signal can only be detected in the STK11IP KO and S404A-STK11IP reconstituted cells, when the pH 4.5 nanoprobe (with a pH threshold of 4.5) was applied (Fig. 3e, f). These results were also consistent with the pH value measured using the lysoSensor yellow/blue dye DND-160 method (Fig. 3c, d).

We further explored the potential mechanism by which STK11IP regulates lysosomal acidification. Lysosomes maintain their pH gradients through the proton-pumping V-type ATPase^47,48^. We, therefore, asked whether STK11IP has an impact on the V-ATPase activity. We isolated the lysosomes from WT and STK11IP KO cells, or WT-, S404A-, and S404D-STK11IP-reconstituted cells, and checked the V-ATPase activity using a re-acidification assay^49^. We found that the activity of V-ATPase was enhanced in the lysosomes isolated from STK11IP KO and S404A-STK11IP cells compared to their control cells (Fig. 3g–j and Supplementary Fig. 4f).

Next, we sought to understand how STK11IP controls the activity of V-ATPase. First, we performed quantitative proteomic analyses (using the tandem mass tag, TMT labeling approach) of STK11IP WT and KO cells. Through these experiments, we found that STK11IP KO has no effect on the expression of the various components of V-ATPase (Supplementary Fig. 5a, Dataset 3). Next, we examined whether STK11IP can interact with the V-ATPase complex. We used the TurboID system to identify the STK11IP-interacting proteins^50^ (Fig. 3k). We observed that many of these biotinylated proteins were localized on the lysosome and were involved in mTORC1 signaling, including the Rag-GTPase, RAPTOR, mTOR, and LAMTOR1. Although STK11IP has been reported to bind LKB1 and regulate TGF-beta signaling^32,33^, we did not identify LKB1 in our TuroID-MS data set, potentially due to the different experimental conditions. Notably, we also identified several components of the V-ATPase complex, including ATP6V1B, ATP6V1A, and ATP6V1H (Fig. 3l and Supplementary Fig. 5b, Dataset 4). The interaction between STK11IP and the various subunits of the V-ATPase complex was further validated by co-IP (Supplementary Fig. 5c–e). Through domain mapping experiments, we further identified that the domain of amino acid 360 to amino acid 770 of STK11IP mediated this interaction between STK11IP and ATP6V1A (Supplementary Fig. 5f).

Interestingly, we found that compared to the WT protein, the S404A-STK11IP mutant formed weaker interaction with the V-ATPase components (ATP6V1A and ATP6V1B) (Fig. 3m and Supplementary Fig. 5g, h). Compared to the WT-STK11IP domain of amino acid 360 to amino acid 770, the same domain bearing the S404A mutation displayed a weaker interaction with ATP6V1A (Fig. 3n). These data indicated that mTORC1-mediated phosphorylation of STK11IP at S404 is essential for its interaction with the V-ATPase complex. Taken together, mTORC1 signaling regulates the phosphorylation of STK11IP at S404. De-phosphorylation of STK11IP at S404 weakened its interactions with V-ATPase. The de-phosphorylation of STK11IP results in increased V-ATPase activity, more acidic lysosomes, enhanced formation of the autolysosomes, and autophagy flux (Fig. 3o).

### STK11IP deficiency protects against fasting- and MCD-induced fatty liver

Autophagy occurs in virtually all cells to maintain cellular homeostasis, including modulation of nutrient balances^42,51^. To investigate the physiological function of STK11IP in mice, we first measured the expression profile of STK11IP in mouse tissues. Results from qRT-PCR and immunoblot analyses showed that *Stk11ip* is widely expressed, with higher expression in the liver, white adipocyte tissue (WAT), and pancreas (Supplementary Fig. 6a, b).

**Fig. 4:**
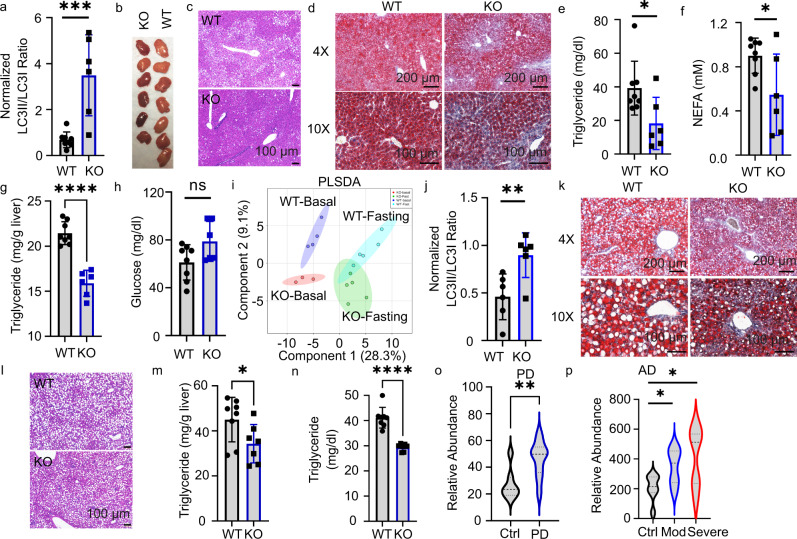
STK11IP deficiency protects mice against metabolic disorder. **a** STK11IP KO leads to increased autophagy in the mice after 36 h of fasting. *WT* wild type, *KO* STK11IP knockout. 3-month-old, WT, *n* = 8; KO, *n* = 6 mice. ***P  = 0.0001, ***P  = 0.0008. **b** Images of the liver from STK11IP WT and KO mice after 36 h of fasting (3-month-old, WT, *n* = 7; KO, *n* = 6 mice). **c**, **d** Histological sections (H&E staining) **c** and Oil-Red staining **d** of the liver from STK11IP WT and KO mice. The mice (4-month-old) were subject to 36 h of fasting after 2-month-HFD diet. (WT, *n* = 10; KO, *n* = 9 mice). Scales bars, 100 μm or 200 μm as indicated. **e**–**h** Blood triglycerides **e**, NEFA (non-esterified fatty acids) **f**, liver triglycerides **g**, and blood glucose **h** levels in the STK11IP WT and KO mice after 36 h of fasting. 3-month-old, WT, *n* = 8; KO, *n* = 6. *P  = 0.03, *P  = 0.0305, ****P < 0.0001, n.s, not significant. **i** Partial least squares discriminant analysis (PLSDA) of the blood metabolites from STK11IP WT or KO mice (3-month-old, WT-basal, *n* = 4; WT-Fasting, *n* = 5; KO-basal, *n* = 3; KO-fasting, *n* = 5 mice). **j** Statistics of the LC3II/LC3I ratio in the liver from STK11IP WT or KO mice (2-month-old) with 2 weeks of the MCD diet. *n* = 6 mice. **P  = 0.0096. **k**, **l** Oil-Red staining (**k**) and histological sections (H&E staining) (**l**) of the liver from STK11IP WT or KO mice after 2 weeks of MCD diet. (WT, *n* = 8; KO, *n* = 7). Scales bars, 100 μm. **m**, **n** Blood triglycerides **m** and liver triglycerides **n** levels in STK11IP WT or KO mice (2-month-old) after 2 weeks of MCD diet. WT, *n* = 8; KO, *n* = 7 mice. *P  = 0.0437, ****P < 0.0001. **o**, **p** STK11IP expression is increased in neurogenerative diseases. STK11IP expression in control individuals, or patients with Parkinson’s disease (**o**: PD) or Alzheimer’s disease (**p**: AD). The data were extracted from the database with a reference number of GSM184355 (PD) and GSM697332 (AD), respectively; (normal: *n* = 7, PD: *n* = 17; *n* = 7 for each group in AD). ****P = 0.0035, *P = 0.0269, *P = 0.0262. All of the statistical results represent mean ± SEM, the statistical significance was calculated with unpaired two-tailed Student’s *t* tests. Source data are provided as a Source Data file.

Several studies have reported that starvation- or exercise-induced autophagy can lead to beneficial metabolic effects^40,52,53^. After 36 h of fasting, we found the phosphorylation of pS404-STK11IP levels decreased (Supplementary Fig. 6c). The STK11IP KO mice showed a robust increase of LC3I to LC3II conversion under these conditions (Fig. 4a and Supplementary Fig. 6d). These results further indicate that STK11IP and its phosphorylation of pS404 could play a role in this process. This enhancement of autophagy significantly improved the fasting-induced fatty liver shown as the decrease of lipid droplets in the STK11IP KO mice (Fig. 4b–d). There was also a concomitant decrease of TG and NEFA (Non-esterified fatty acids) levels in serum and liver (Fig. 4e–g). STK11IP KO mice also exhibited relatively higher serum glucose levels compared to WT mice, although this difference was insignificant (Fig. 4h). These observations are consistent with the previous report that autophagy promotes the conversion of lipids to glucose during long-term fasting^54^.

To gain further insights into the metabolic remodeling after STK11IP KO, we performed metabolomic profiling of the serum harvested from WT and STK11IP KO mice. Partial least squares discriminant analysis (PLSDA) showed distinct metabolic patterns for the WT and STK11IP KO groups under both basal and fasted conditions (Fig. 4i and Supplementary Fig. 6e, Dataset 5). Quantitative pathway enrichment analyses revealed a significant enrichment of glucogenesis, ketone body metabolism, beta-oxidation of fatty acids, and betaine or carnitine metabolism (Supplementary Fig. 6f). These alterations may explain why the glucose level was higher and the TG level was lower in STK11IP KO mice after long-term starvation (Fig. 4e–h). This is consistent with previous reports that upon starvation, fatty acids and amino acids generated by autophagy can be used to drive gluconeogenesis and ketogenesis. As starvation continues, degradation of adipose and muscle tissues plays an increasing role in supplying substrates to the liver, which exports glucose and ketone bodies to feed the other tissues^54^.

Metabolic perturbations, including insulin resistance and altered lipid metabolism, have been hypothesized to contribute to the pathogenesis of NASH, which is characterized by the imbalance in triglyceride (TG) production, uptake, and removal in the liver. Mechanisms that promote fat clearance are expected to reduce NASH. We, therefore, utilized a NASH model that is induced by the Methionine-Choline-deficient (MCD) diet^55–57^. After 2 weeks of the MCD diet, STK11IP KO mice showed a similar decrease in body weight, compared with WT mice (Supplementary Fig. 6g). However, compared to control cells, the liver from STK11IP KO mice showed increased LC3II/LC3I ratios (Fig. 4j and Supplementary Fig. 6h). Consistent with this, STK11IP KO mice showed reduced liver steatosis, as indicated by the reduced macro-vesicular lipid droplets (Fig. 4k, l) and the downregulation of TG levels in the serum and liver tissue (Fig. 4m, n).

## Discussion

mTORC1 functions at the convergence point of a vast signaling network that senses fluctuations in extracellular and intracellular nutrients to regulate a large variety of cellular anabolic processes^58^. The activation of mTORC1 is mainly achieved by two conditions, the nutrient-dependent activation of the heterodimeric Rag-GTPases that recruit mTORC1 to the lysosomal surface, together with the growth factor-driven activation of the lysosome-bound Rheb-GTPase^3^. Once mTORC1 is activated, whether mTORC1 phosphorylates local lysosomal proteins to mediate its downstream signaling output, however, is poorly understood. To identify the local lysosomal substrates of mTORC1, we combined and cross-referenced two datasets, i.e., a lysosomal proteomic dataset (Fig. 1a) and our previous hepatic mTORC1-regulated phosphoproteomic datasets^13^. These integrative analyses led to the identification of a poorly studied protein, STK11IP^33^, as a potential lysosomal mTORC1 substrate (Fig. 1b, c). Further biochemical studies showed that: (1) STK11IP is localized on the lysosomal membrane, (2) the phosphorylation level of STK11IP at S404 correlates with the activity of mTORC1 in cells, (3) STK11IP interacts with Raptor, but not Rictor, and (4) STK11IP-S404 is phosphorylated by mTOR in vitro. These results indicate that STK11IP is a novel, lysosome-localized substrate of mTORC1. In addition to STK11IP, our database also contains many lysosomal proteins, as well as other potential non-lysosomal mTORC1 substrates that will be useful for future research focused on lysosomal biology and mTORC1 signaling (Fig. 1b). Finally, the integration of organelle proteomic and global quantitative phosphoproteomic results offers a general strategy for the identification of the organelle-specific substrates for a kinase of interest.

Autophagy is a self-digestion process that is critically involved in the elimination of defective/accumulated proteins and damaged organelles, as well as the removal of intracellular pathogens. The dysregulation of autophagy is implicated in the pathogenesis of a broad range of diseases, including aging, metabolic syndrome, neurodegenerative disease, cancer, and heart disease^51^. Lysosomes, which are known as the cellular “incinerators”, not only provide the platform for mTORC1 activation but also receive signals from mTORC1 to regulate autophagy. We showed that cells with STK11IP KO had increased autophagy flux. In addition to this, STK11IP could also regulate other aspects of autophagy biology, and these will be addressed in future studies. Besides STK11IP, TFEB is another mTORC1 substrate that is also localized on the lysosome^17,18,59^. Our data revealed a novel, lysosomal acidification-related mechanism by which mTORC1 regulates autolysosome formation and autophagy (Fig. 3o). We found that STK11IP phosphorylation could regulate V-ATPase activity through the interaction of STK11IP with ATP6V1A (mediated by the domain of 360–770 of STK11IP (Supplementary Fig. 5f)). How STK11IP and V-ATPase interactions regulate the activity of V-ATPase, however, will be addressed by future structural studies of this protein complex. Finally, a recent study showed that the luminal pH values of individual lysosomes are unequal, and this heterogeneity could result from the position of each lysosome within the cell^60^. Our data thus raise the intriguing hypothesis that the distribution of activated mTORC1 in cells and hence pS404-STK11IP status could be a mechanism to explain the position-dependent regulation of lysosomal acidification.

We explored the in vivo implications of our findings using a STK11IP KO mouse model. During starvation, autophagy in the liver generates nucleosides, fatty acids, and amino acids to provide the substrates for ketogenesis and glucogenesis. Inhibition of autophagy will result in more lipids storage in the liver^54,61^. In this study, we observed that STK11IP KO mice showed enhanced autophagy. Under starvation conditions, the serum metabolites that were differentially present between WT and STK11IP KO mice were mainly linked to ketone body metabolism and glucogenesis (Supplementary Fig. 6e, f). As a result, STK11IP KO mice exhibited a lower serum TG and a higher blood glucose level relative to their controls, which is consistent with enhanced autophagy in STK11IP KO mice (Fig. 4e–h). In addition, metabolic perturbations, including insulin resistance and impaired lipid metabolism, have been hypothesized to contribute to the pathogenesis of NASH (nonalcoholic steatohepatitis). Autophagy is known to be deregulated during the pathogenesis and progression of NASH. Impaired autophagy prevents the clearance of excessive lipid droplets. Rapamycin treatment or FNDC5 KO results in enhanced autophagy, which can ameliorate NASH^62,63^. Indeed, we found that STK11IP KO can reduce MCD diet-induced NASH in mice (Fig. 4j–n and Supplementary Fig. 6h).

Because autophagy also plays a critical role in neurodegenerative diseases, we also searched public databases and found that STK11IP expression is upregulated during the progression of neurodegenerative diseases, including Parkinson’s disease and Alzheimer’s disease (Fig. 4o, p). Intriguingly, using a large-scale, multi-omics profiling approach, ATP6V1A (one of the STK11IP-interacting proteins) has recently been identified as a key regulator of the pathogenesis of sporadic late-onset Alzheimer’s disease^64^. All these results point to the possibility that STK11IP could also be involved in the regulation of autophagy implicated in neurodegeneration. It is important to note that STK11IP depletion has no effect on the activity of mTORC1. Agents that pharmacologically target STK11IP therefore will offer a strategy to manipulate autophagy, avoiding the known side effects associated with mTORC1 inhibitors (e.g., rapamycin)^65–69^. The full therapeutic potential of these agents in the treatment of the relevant diseases will be addressed in future studies.

In summary, we used quantitative proteomics and identified STK11IP as a novel, lysosomal-specific substrate of mTORC1. STK11IP and its mTORC1-mediated phosphorylation regulate lysosome acidification and autolysosome maturation. Although the detailed mechanism about how STK11IP regulates autolysosome formation through regulating the lysosome acidification is not clear, this offers a novel, TFEB/ULK1-independent mechanism for mTORC1 to regulate autophagy. STK11IP ablation leads to enhanced autophagy in mice, which protects these animals against NASH. This study provides the foundation for targeting STK11IP for the treatment of diseases with aberrant autophagy signaling.

## Methods

### Materials

All the materials including antibodies, plasmid, and reagents were listed in Supplementary Dataset 6.

### Cell culture

HEK293T (ATCC, Cat# CRL-3216), Hela (ATCC, Cat# CCL-2) and primary MEF cells (isolated from wild type and STK11IP KO mice) were cultured in Dulbecco’s modified Eagle’s medium (DMEM, Sigma, D5796) supplemented with 10% fetal bovine serum (FBS, Sigma, 12306 C) and antibiotics (Gibco, 15240112) at 37 °C in a 5% CO_2_ incubator.

All the stable cells, including STK11IP KO cells, reconstituted cells, or GFP-LC3-RFP stable cells were screened for single-cell clones using FACS and were confirmed using western blotting analyses.

### LC3 turnover assay

WT and STK11IP KO HEK293T cells were cultured in regular DMEM with or without 2 h of 20 μM CQ or 100 nM Bafilomycin A1 treatment. The cells were lysed using the lysis buffer (1% sodium dodecyl sulfate (SDS), 10 mM HEPES, pH 7.0, 2 mM MgCl_2_, 20 U ml^−1^ universal nuclease). The same amounts of proteins were subject to immunoblotting analyses. Quantification of western blots was performed using Image J 1.50i.

### Mice and treatment

Wild-type (005304), STK11IP KO mice (028999), and RFP-GFP-LC3 transgenic mice (027139) were purchased from the JAX lab. All experimental animals were housed in a barrier-specific pathogen-free animal facility with 12 h light/12 h dark cycle with free access to water and food.

Mice were genotyped followed the protocol provided by the JAX lab. STK11IP KO mice were genotyped using primers (533:CAGTGTGCTACAGCCAGAGAG;532:GAGCTGGGGAGGAGGTAGAC;534:AGGCCATCTCTCTGTCCTCA). mRFP-GFP-LC3 mice were genotyped using primers (24935:CATGGACGAGCTGTACAAGT;24936:CACCGTGATCAGGTACAAGGA; oIMR7338:CTAGGCCACAGAATTGAAAGATCT;oIMR7339GTAGGTGGAAATTCTAGCATC:ATCC).

Mice were maintained on a 12-h dark/12-h light cycle and housed in groups of 3–5 with unlimited access to water and food. The normal chow diet was LabDiet (5058); high-fat diet (60% fat, D12451) and MCD diet (methionine and choline deficiency diet, A02082002BR) were purchased from Research Diet. The Institutional Animal Care and Use Committee of the University of Texas Southwestern Medical Center approved all animal experiments. All efforts were made to follow the Replacement, Refinement, and Reduction guidelines. To minimize discomfort, mice were anesthetized with a ketamine/xylazine cocktail or 2–3% isoflurane during surgery or before organ harvest. The STK11IP KO mice were backcrossed to the 6th generation with wild-type C57BL/6NJ mice when performing the experiments.

### TG, NEFA, cholesterol measurement

Blood samples were collected from the tail vein. The same amount of liver tissue samples were collected. The blood or liver triglycerides, blood cholesterol, and NEFA levels were measured following the manufacturer’s instructions.

### Isolation of the lysosomes

Confluent HEK293T cells that stably expressing LAMP1-GFP-FLAG^X3^ (LGF) or STK11IP-GFP-Flag^X3^ (SGF) were rinsed with cold PBS, scraped, spun down, and resuspended in 750 μl of fractionation buffer: 140 mM KCl, 5 mM MgCl_2_, 50 mM Sucrose, 20 mM HEPES, pH 7.4, supplemented with protease inhibitors. The cells were mechanically broken with a pellet pestle and were spun down at 1000 × *g* for 10 min to remove the nucleus and cell debris. The samples were then spun down at 20,000 × *g* for 25 min to yield the organelles pellets. The pellet was resuspended in the fractionation buffer and subject to IP with 100 μl of a 50% slurry of anti-FLAG magnetic beads (M8823, Sigma). Four hours later, beads were washed four times using the fractionation buffer, then eluted with 1% SDS lysis buffer (1% SDS, 10 mM HEPES, pH 7.0, 2 mM MgCl_2_, 20 U ml^−1^ universal nuclease), and the proteins were subjected to immunoblot or MS analysis.

### RNA extraction and qRT-PCR

Cells or tissues were lysed in the TRIzol reagent (Sigma, T9424), and mRNA was extracted according to the standard protocols. Reverse transcription was performed using the SuperScript™ III One-Step RT-PCR System (Invitrogen, 12574026) according to the manufacturer’s instructions. qRT-PCR was performed with a real-time system (SYBR green, Applied Biosystems, A25741) using the qRT-PCR machine (BioRad, CFX Manager 3.0). Data were normalized to the internal control and presented as relative expression levels.

### Co-immunoprecipitation

HEK293T cells were rinsed once with ice-cold PBS after the transfection (24–36 h and lysed in the ice-cold BLB lysis buffer (10 mM KPO_4_, pH 7.6, 6 mM EDTA, pH 8.0, 10 mM MgCl_2_, 0.5% NP-40, 0.1% BriJ-35, 0.1% DOC, adjust pH to 7.4) or IP buffer (0.5% NP-40, 150 mM NaCl, 50 mM Tris-HCl, pH 7.5) added with EDTA-free protease inhibitors (Roche). The soluble fractions from cell lysates were isolated by centrifugation at the highest speed for 10 min. For IPs, 20 μl of a 50% slurry of anti-FLAG (A2220, Sigma) or anti-HA beads (A2095, Sigma) were added to each lysate and incubated with rotation overnight at 4 °C. Immunoprecipitants were washed three times with buffer. Immunoprecipitated proteins were denatured by the addition of 100 μl of sample buffer and boiling for 10 min and subject to immunoblot analysis.

### Histology and immune staining

Tissues were fixed in 4% paraformaldehyde (PFA) and were embedded in paraffin or frozen sections. Paraffin sections (5 µm) were deparaffinized and the frozen sections were washed with PBS to remove the OCT compound. The hematoxylin (Vector, H3401) and eosin Y (Thermo, 6766007) staining were performed according to standard protocols or the manufacturer’s instructions.

Cells were plated on micro cover glasses (Electron Microscopy Sciences 72226-01). After confluence, cells were washed with PBS and fixed with 4% PFA in PBS for 10 min. The cells were permeabilized (0.2% Triton X-100, 10 min), blocked in 3% BSA (Sigma, A9418), then incubated with primary antibodies overnight at 4 °C (for example, the LC3, LAMP2, Tom20, and Rab7 antibodies). After the incubation with primary antibodies, slides were washed and incubated with Alexa Fluor 546 goat anti-rabbit or Alexa Fluor 488 goat anti-mouse secondary antibodies (Life Technologies, A11001, and A11010) at room temperature for 1 h. The slides were then washed and stained with DAPI (Sigma, D9542), and sealed with the mounting medium (FluorSave reagent, Millipore, 345789). Photomicrographs were taken of representative ×10, ×20, or ×40 magnification fields using a confocal microscope (Confocal Zeiss LSM880 Airyscan, Zeiss Zen).

### Flow cytometry

Cells were washed, trypsinized, and centrifuged at 400 × *g* for 4 min. Cells were washed once with FACS buffer (DPBS with 2% FBS), then resuspended in the FACS buffer for FACS analysis (LSRFortessa, BD) or sorting (FACS Aria II SORP, BD). The figure exemplifying the gating strategy was shown in Supplementary Fig. 7.

### Immunoblot analysis and subcellular fraction

For immunoblot analysis, the cells were lysed using the 1% SDS lysis buffer (1% SDS, 10 mM HEPES, pH 7.0, 2 mM MgCl_2_, 20 U l^−1^ universal nuclease with added protease inhibitors and phosphatase inhibitors). Protein concentrations were measured using the BCA assay (23227, Thermo Fisher). The lysates were mixed with the 4× reducing buffer (60 mM Tris-HCl, pH 6.8, 25% glycerol, 2% SDS, 14.4 mM 2-mercaptoethanol, 0.1% bromophenol blue). Samples were boiled for 10 min and the same amounts of proteins were subjected to electrophoresis using the standard sodium dodecyl sulfate–polyacrylamide gel electrophoresis method.

Proteins were then transferred to a 0.22 μm nitrocellulose membrane (GE, 10600001), and were blocked with a TBST buffer (25 mM Tris-HCl, pH 7.5, 150 mM NaCl, 0.05% Tween-20) containing 3% non-fat dried milk and incubated overnight with primary antibodies (1:1000 dilution) at 4 °C and for 1 h at room temperature with peroxidase-conjugated secondary antibodies (1:10,000 dilution). Blots were developed using enhanced chemiluminescence, were exposed on autoradiograph films, and were developed using standard methods.

All the western blot images were quantified by using the software package Image J 1.50i.

### Isolation of primary MEF cells

MEF were isolated from wild-type and STK11IP KO mice or WT/STK11IP KO mice crossing with mRFP-GFP-LC3 mice following the previous protocol^70^.

### Endurance exercise test

For the endurance exercise test, mice (wild type and STK11IP KO mice crossed with or without mRFP-GFP-LC3) were acclimated to and trained on an exercise treadmill (Columbus Instruments) for 2 days. On day 1, the mice ran for 5 min at 8 m min^−1^ and on day 2, the mice ran for 5 min at 8 m min^−1^ followed by another 5 min at 10 m min^−1^. On day 3, the mice were subjected to a single bout of running starting at the speed of 10 m min^−1^. Forty minutes later, the treadmill speed was increased at a rate of 1 m min^−1^ every 10 min for a total of 30 min, and then increased at the rate of 1 m min^−1^ every 5 min until mice were exhausted (Shown in Supplementary Fig. 3i). Exhaustion was defined as the point at which mice spent more than 5 s on the electric shocker without attempting to resume running. Total running time was recorded, and total running distance was calculated for each mouse^40,71^.

### Metabolomics

For metabolites extraction, ice-cold methanol was added to the blood samples to a final concentration of 80%. Metabolite profiling was performed with LC–MS/MS. The peak area for each detected metabolite was normalized to the total ion count. The preprocessed data sets were mean-centered, and unit-variance scaled. Principal component analysis and hierarchical clustering of metabolites in different samples were performed using MetaboAnalyst 5.0.^72^.

### Biotin labeling with TurboID in HEK293T cells

The TurboID-STK11IP stably expressing HEK293T cells were cultured in no-Biotin DMEM medium for five generations. Biotin (50 μM) labeling was then initiated with complete or amino acid depleted medium for 2 h. The labeling was stopped by washing the cells with ice-cold PBS and the cells were lysed with the 1% SDS lysis buffer. The lysates were diluted to 0.1% SDS with PBS, and the proteins were immunoprecipitated using streptavidin beads. After the pull-down, beads were washed three times, and were boiled for 30 min. The eluate was subject to immunoblot. For the MS sample, which was prepared using on-beads digestion following the previous report^50^.

### Mass spectrometry experiment

Protein lysates were reduced by 2 mM dithiothreitol, and alkylated by adding iodoacetamide to a final concentration of 50 mM, followed by incubation in the dark for 20 min. The lysates were extracted by methanol–chloroform precipitation and the proteins were solubilized in 8 M urea, then digested with Lys-C. The samples were then diluted to a final concentration of 2 M urea by the addition of 100 mM ammonium bicarbonate (pH 7.8). Proteins were digested overnight with sequencing-grade trypsin at a 1:100 (enzyme/substrate) ratio. Digestion was quenched by the addition of trifluoroacetic acid to a final concentration of 0.1%. Peptides were desalted using SepPak C18 columns (Waters) according to the manufacturer’s instructions. Peptides were fractionated by using an off-line two-dimensional SCX-RP-HPLC (strong-cation-exchange reversed-phase HPLC) protocol.

The MS samples were analyzed by LC–MS/MS experiments on an LTQ Velos Pro Orbitrap mass spectrometer (Thermo) using a top-20 CID (collision-induced dissociation) method. MS/MS spectra were searched against a composite database of the human protein database using the Proteome Discovery and its reversed complement using the Sequest algorithm. Search parameters allowed for a static modification of 57.02146 Da for cysteine and a dynamic modification of oxidation (15.99491 Da) on methionine, respectively. Search results were filtered to include <1% matches to the reverse database by the linear discriminator function using parameters including Xcorr, dCN, missed cleavage, charge state, mass accuracy, peptide length, and a fraction of ions matched to MS/MS spectra. Peptide quantification was performed by using the Core Quant algorithm^73^.

For the data process of lysosome proteomics, we used the LysoIP-proteomics experiments to define the lysosomal proteome. For these analyses, we performed two biological replicate experiments. After the MS experiments, we performed cross-reference analyses and excluded the proteins that were commonly identified in both the control (GFP-Flag) and the LGF (LAMP1-GFP-Flag) sample. These excluded proteins were classified as non-specific proteins. After filtering out these non-specifically bound proteins, we were able to derive a list of proteins that were specifically identified in the LGF sample. To increase the confidence in the protein identification, we also only selected the proteins that were identified with at least two unique peptides.

### LysoSensor/LysoTracker staining and pH measurement

Wild-type/STK11IP KO cells or WT/S404A/S404D reconstituted cells were plated in eight-well charmers containing 200 µL complete DMEM medium. In a typical procedure, the cells were cultured in a complete medium, then 25 µg/mL of TMR-conjugated hybrid nanoprobes were added and kept for 15 min at 37 °C. The medium was removed and washed. Thereafter, cells were incubated with a complete medium for 1 h at 37 °C. The TRITC (590 ± 25 nm) filters were used for TMR imaging.

For LysoTracker or LysoSensor imaging, the cells were incubated in a complete DMEM medium, then LysoTracker® Red DND-99 (100 nM) and LysoSensor (2 µM) was added for one hour, then the fluorescence was monitored using confocal.

For pH value measurement, quantification of lysosomal pH was performed using a ratiometric lysosomal pH dye LysoSensor Yellow/Blue DND-160. The pH calibration curve was generated as described previously^74^. In brief, cells were labeled with 2 µM LysoSensor Yellow/Blue DND-160 for 30 min at 37 °C in regular medium, and excessive dye was washed out using PBS. The labeled cells were treated for 10 min with 10 µM monensin and 10 µM nigericin in 25 mM MES calibration buffer, pH 3.5, 4.0, 4.5 5.0, 6.0, and 7.0, containing 5 mM NaCl, 115 mM KCl, and 1.2 mM MgSO_4_. Quantitative comparisons were performed in a 96-well plate, and the fluorescence was measured with a microplate reader (Microplate Manager 3.05.11) at 37 °C. Light emitted at 440 and 535 nm in response to excitation at 340 and 380 nm was measured, respectively. The ratio of light emitted at 440 and 535 was plotted against the pH values in MES buffer, and the pH calibration curve for the fluorescence probe was generated from the plot and the result was analyzed using GraphPad Prism 9.

### Ultra-pH-sensitive nanoparticles staining

The TMR4.5 (4.4) nanoprobe was chosen from the library of Ultra-pH-sensitive nanoparticles as presented in ref. ^46^, with *x* = 0, *y* = 80, R2 = Pentyl and Dye = TMR^46^. This probe has a sharp pH response across the pH threshold (pHt) of 4.4–4.5, which shows minimal fluorescent signal when environment pH is above pHt while ~100 folds increased signal when the pH is below pHt^46^. The lysosome-colocation of UPS nanoparticles (including 4.4-TMR) was validated in^75^ by co-staining of LAMP2 and Rab5. These Ultra-pH-sensitive nanoparticles were used to detect lysosome pH values in the previous report^45^.

### Lysosome re-acidification assay

The lysosomes of wild type/STK11IP−/− or WT/S404A/ S404D reconstituted HEK293T cells were loaded with LysoSensor™ Yellow/Blue DND-160, and performed following published procedures^49^. The cells were washed with a buffer (90 mM K-gluconate, 50 mM KCl, 1 mM EGTA, 20 mM HEPES, 50 mM sucrose, and protease inhibitor cocktail, pH 7.3) before the treatment with 1 µM FCCP for 15 min in order to completely dissipate the lysosomal pH gradient. The FCCP-treated cells were harvested and homogenized using a Dounce homogenizer. To prevent lysosomal re-acidification during the course of fractionation, the cells were maintained in the continued presence of FCCP at 4 °C, until the final wash of the isolated organelles. The organelles were resuspended in 1 ml buffer without FCCP. The fluorescence was monitored using a plate reader (Microplate Manager 3.05.11) (emission at 440 and 535 nm in response to excitation at 340 and 380 nm, respectively). After the baseline determination (4 min), 5 mM MgCl_2_ and 5 mM ATP were added to the suspension, and fluorescence was monitored for 20 min. The ratio of light emitted at 440/535 was calculated and the result was analyzed using GraphPad Prism 9.

### Transmission electron microscopy

For electron microscopy (EM) studies, cells were plated on glass-bottom MatTek dishes (MatTek, P35G-1.5-14-C) and fixed with EM fixation buffer (2.5% glutaraldehyde, 0.1 M phosphate buffer pH 7.4), followed by 1% OsO4. The cells were further dehydrated, cut into thin sections, and stained with uranyl acetate and lead citrate. All the images were obtained using an EM JEOL 1400 Plus electron microscope with a digital camera.

### In vitro kinase assay

STK11IP was phosphorylated by mTOR in vitro. HEK293T cells that stably express Flag-STK11IP were treated with Torin1 (2 μM) for 6 h, and Flag-Tagged STK11IP was pulled down from these cells. Recombinant mTOR was purchased from Millipore (14-770) and recombinant 4EBP1 was purchased from Millipore (516678). Flag-STK11IP or 4EBP1 was incubated with or without mTOR in 50 μl reaction mixtures at 37 °C for 60 min. The reaction mixture contained 1× mTOR kinase buffer (Invitrogen), 1× protease inhibitor cocktail (Roche), 2 mM DTT, 10 μM ATP, 1 μg Flag-STK11IP, and 250 ng mTOR. The reaction was stopped by adding 16 μl of 4× SDS sample buffer. The samples were boiled at 95 °C for 5 min, and were then subject to immunoblot analysis^11^.

### Statistical analysis and reproducibility

All the experiments were repeated three times, with similar results. The representative results were shown in the manuscript. All of the statistical analyses (unpaired, two-tailed *t* tests) were performed using the GraphPad Prism software 9. Data were derived from the average of replicate experiments (indicated in the figure legends) and calculated as mean ± SEM. *P < 0.5, **P < 0.01, ***P < 0.001, ****P < 0.0001, n.s, not significant.

### Reporting summary

Further information on research design is available in the Nature Research Reporting Summary linked to this article.

## Supplementary information

Supplementary Information

Description of Additional Supplementary Files

Supplementary Data 1

Supplementary Data 2

Supplementary Data 3

Supplementary Data 4

Supplementary Data 5

Supplementary Data 6

Reporting Summary

## Data Availability

The mass spectrometry proteomics data have been deposited to the ProteomeXchange Consortium via the PRIDE [1] partner repository with the data set identifier PXD031543, including the raw mass spectrometry proteomic files related to Supplementary Dataset 1, Supplementary Dataset 3 and Supplementary Dataset 4. The source data are provided as a Source Data file. Fully uncropped versions of all gels and blots are shown in Supplementary Fig. 8. A reporting summary for this Article is available as a Supplementary Information file. Source data are provided with this paper.

## Source data

Source Data
