# Supplementary material for: Quantitative phosphoproteomic analyses identify STK11IP as a lysosome-specific substrate of mTORC1 that regulates lysosomal acidification
Source: Nat Commun. 2022 Apr 1;13:1760. doi: 10.1038/s41467-022-29461-8 (PMC8976005; doi:10.1038/s41467-022-29461-8)
Supplement: Supplementary file 1 — Supplementary Information [file 41467_2022_29461_MOESM1_ESM.pdf]

Quantitative Phosphoproteomic Analyses Identify STK11IP as a Lysosome-Specific Substrate of mTORC1 that Regulates Lysosomal Acidification

Zhenzhen Zi[1], Zhuzhen Zhang[2], Qiang Feng[3], Chiho Kim[1], Xu-Dong Wang[1], Philipp E. Scherer[2,4], Jinming Gao[3], Beth Levine[5], Yonghao Yu[1, *]

1. Department of Biochemistry, University of Texas Southwestern Medical Center, Dallas, TX, 75390, USA.
2. Touchstone Diabetes Center, University of Texas Southwestern Medical Center, Dallas, TX, 75390, USA.
3. Harold C. Simmons Comprehensive Cancer Center, University of Texas Southwestern Medical Center, Dallas, TX, 75390, USA.
4. Department of Cell Biology, University of Texas Southwestern Medical Center, Dallas, TX, 75390, USA
5. Howard Hughes Medical Institute and Center for Autophagy Research, University of Texas Southwestern Medical Center, Dallas, TX, 75390, USA

* Correspondence should be addressed to Y.Y.

E-mail: Yonghao.Yu@UTsouthwestern.edu,

Telephone: 214-648-3535

This PDF file includes:

Supplementary Figures 1-8

Supplementary Figures

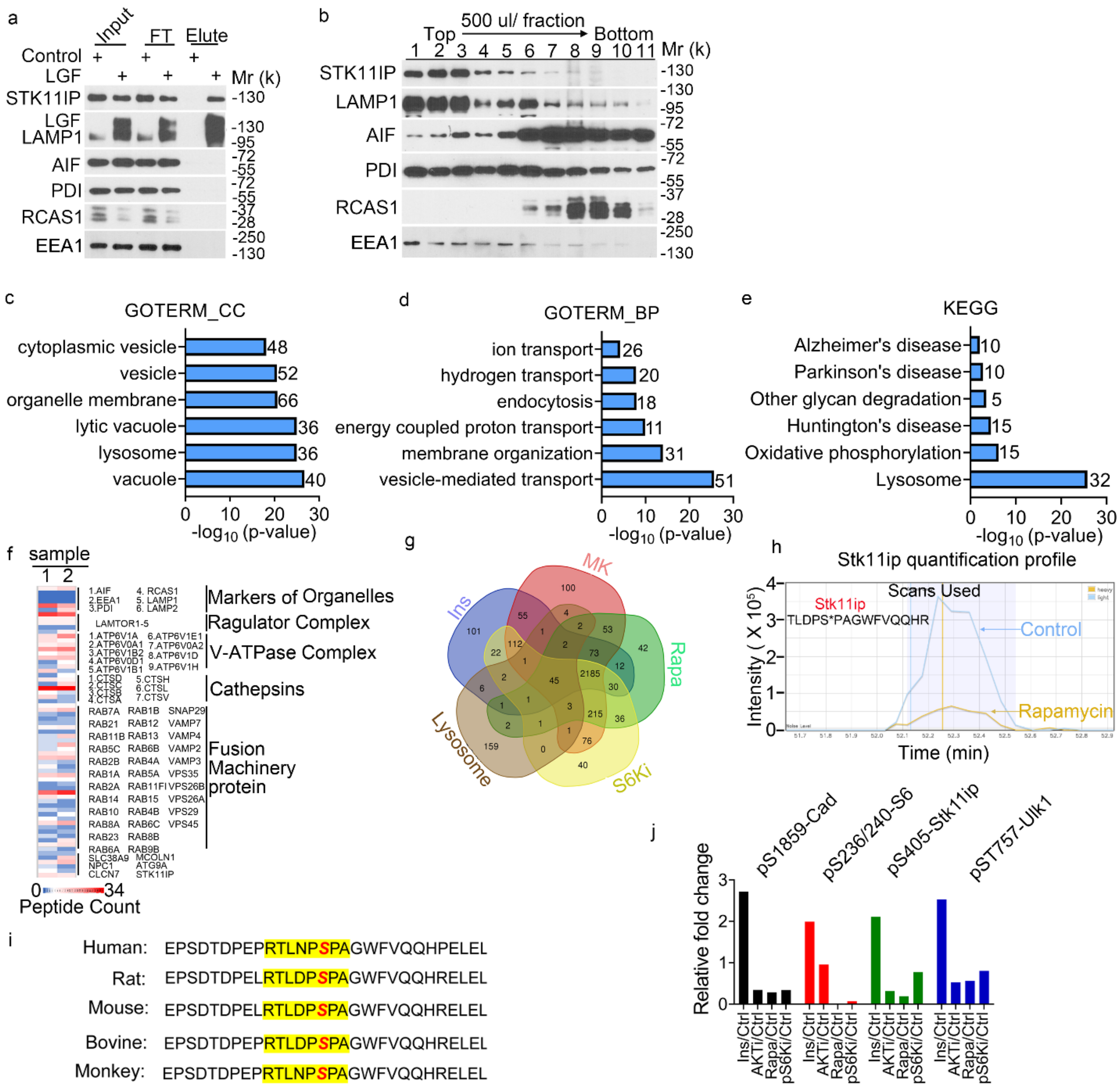

Supplementary Fig. 1. Integrative analyses of the mTORC1-regulated lysosomal phosphoproteome

(a) Immunoblot analyses of the immunopurified lysosomes. The endogenous markers for several organelles were used, including LAMP1 (a lysosome marker, Lyso), AIF (a mitochondrial marker, Mt), PDI (an endoplasmic reticulum marker, ER), RCAS1 (a Golgi Complex marker, Golgi) and EEA1 (an early endosome marker). Mr (k) indicates the molecular weight markers.

(b) Immunoblot analysis of the ultracentrifugation-purified lysosomes.

(c-e) Gene Ontology (GO) analyses of the proteins identified in the lysosome proteomic experiment. The following enrichment studies were performed: biological processes (BP) (c), cell component (CC) (d) and the KEGG pathway (e).

(f) The relative abundances of the known lysosome proteins (data from two biological replicate experiments).

(g) A Venn diagram demonstrating the proteins that were commonly identified between the hepatic phosphoproteomic experiments and the lysosomal proteomic experiments. Ins (Insulin); MK (MK2206, Akt inhibitor); Rapa (rapamycin, mTORC1 inhibitor); S6Ki (S6 kinase inhibitor).

(h) The extracted ion chromatogram of the light (control, blue) and heavy (rapamycin-treated, yellow) ions of a Stk11ip peptide in the rat protein (TLDPS*PAGWFVQQHR, * indicates the phosphorylation site).

(i) pS404-STK11IP is evolutionarily conserved among the various species. The S404 (in human) site is highlighted in red.

(j) The level of changes of several representative phosphopeptides under the indicated conditions, including pS1859-Cad, pS236/240-Rps6, pS405-Stk11ip (pS404 in the human protein) and pT757-Ulk1. The data were extracted from the hepatic phosphoproteomic results.

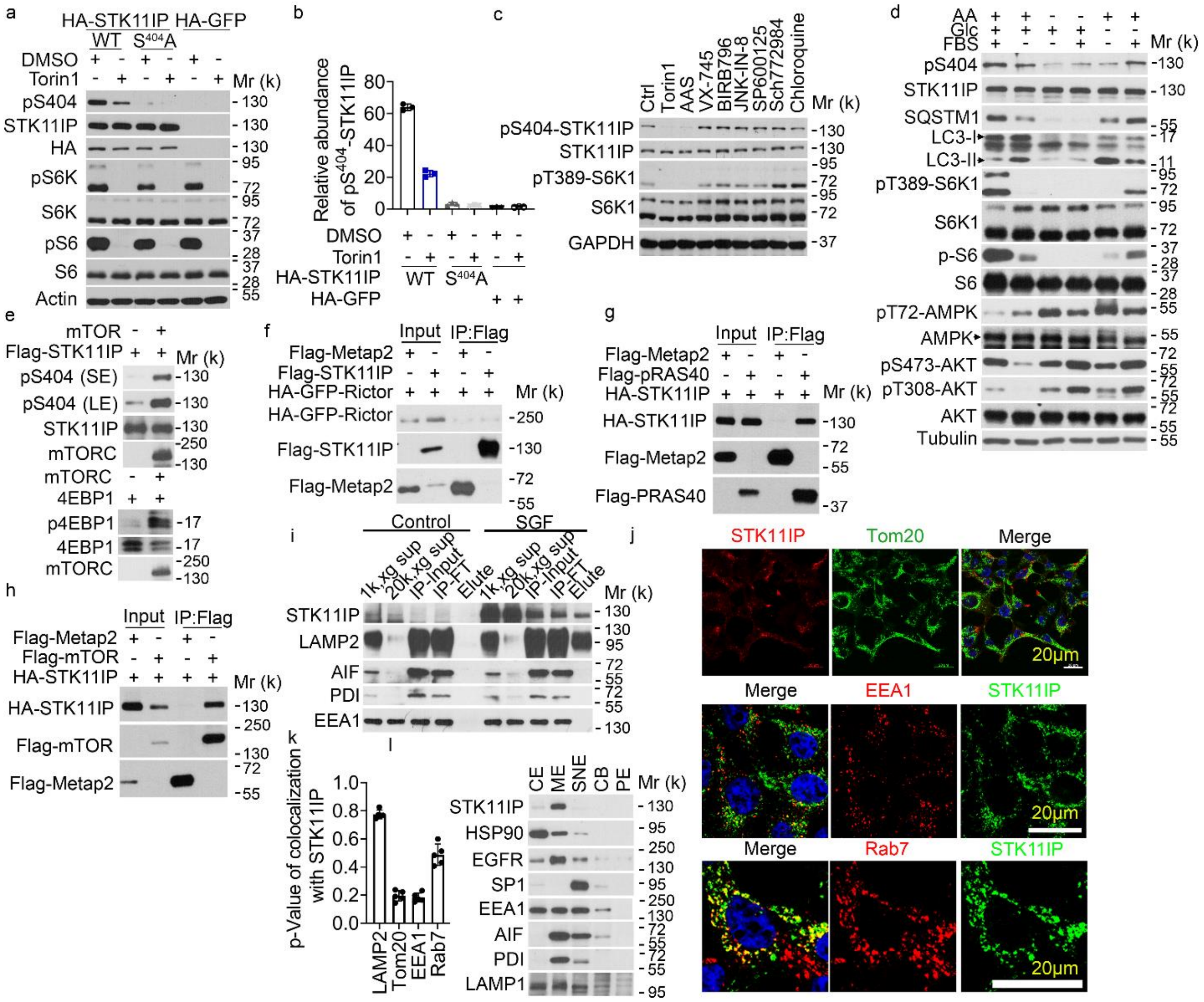

Supplementary Fig. 2. STK11IP is a lysosome-specific mTORC1 substrate.

(a) The antibody against to pS404-STK11IP was specific to pS404-STK11IP. When indicated, the cells were treated with DMSO (Ctrl) or Torin1 (2 μM for 4 hours). STK11IP was immunoprecipitated and was analyzed by the indicated antibodies. The level of pS6K, S6K, pS6, S6 and actin (in the whole cell lysates) was also analyzed.

(b) Quantification of the pS404-STK11IP level from 2a. Data are mean ± SEM. n=3 biologically independent samples.

(c) Immunoblot analysis of pS404-STK11IP levels in HEK293T cells under the indicated treatment conditions for 4 hours. Ctrl (control, DMSO), AAS (amino acid starvation), Torin1 (2 μM), VX-745 (10 μM), BIRB796 (10 μM), JNK-IN-8 (10 μM), SP600125 (10 μM), Sch772984 (10 μM) and CQ (20 μM).

(d) Phosphorylation of STK11IP at S404 is blocked as a result of nutrient deprivation. HEK293T cells were starved for the indicated nutrients (overnight starvation). AA = amino acid. Glc = glucose. FBS = serum.

(e) STK11IP is phosphorylated by mTOR in vitro. 4EBP1 was used as the positive control. SE, short exposure; LE, long exposure.

(f-h) STK11IP does not interact with Rictor (f), but interacts with PRAS40 (g) and mTOR (h).

(i) Immunoblot analysis of the various organelle markers from the STK11IP-GFP-Flag (SGF)-immunopurified lysosomes.

(j) Co-immunofluorescence analyses of STK11IP and Tom20 (a mitochondrial marker, top row), EEA1 (an early Endosome marker, middle row) and Rab7 (an endosome, autophagosome and lysosome marker, bottom row) in the HEK293T cells. Scales bars, 20 μm.

(k) Quantification of the colocalization between STK11IP and LAMP2 (Fig. 1f), Tom20, EEA1 and Rab7 (Supplementary Fig. 2j). The quantification was performed using the Image J 1.50i and was determined using the Pearson correlation of the colocalization coefficient. Data are mean ± SEM, n= 4 independent biology samples.

(l) STK11IP expression in different subcellular fractions. HSP90, a cytoplasmic marker (CE, cytosol fraction); EGFR, a marker of the membrane (ME, membrane fraction); SP1, a marker for the soluble nuclear fraction (SNE); CB (chromatin-bound fraction) and PE (pellet fraction).

Source data are provided as a Source Data file.

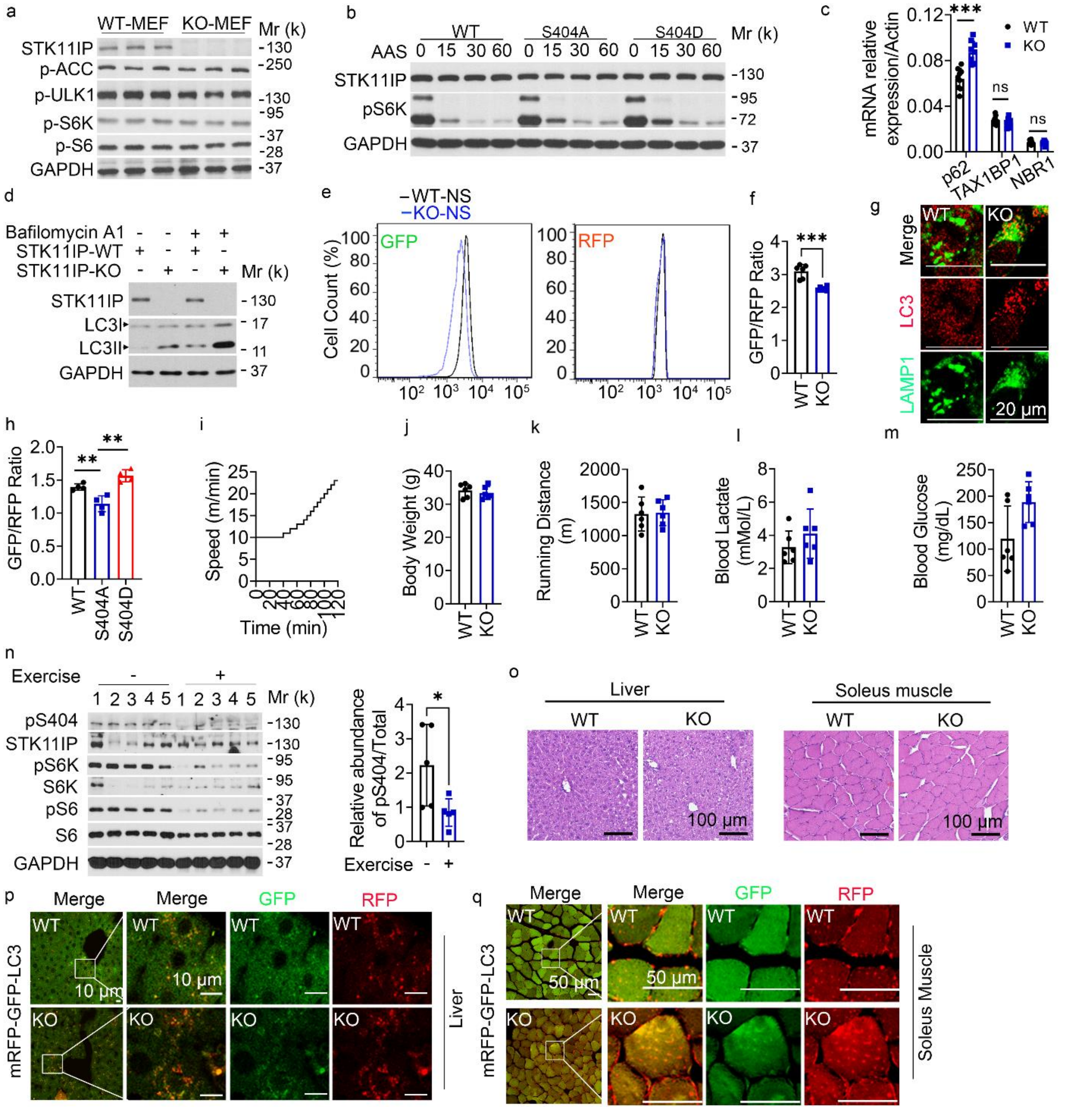

Supplementary Fig. 3. STK11IP Deficiency Promotes Autophagy

(a) STK11IP KO does not affect mTORC1 signaling. WT, wild type; KO, STK11IP knock out.

(b) STK11IP-S404 phosphorylation status does not affect mTORC1 activity.

(c) The mRNA levels of p62 (SQSTM1) increased, while mRNA levels of TAX1BP1 and NBR1 has no change in the STK11IP-KO HEK293T cells compared to the control cells. Data are mean ± SEM, n=3 biologically independent samples (with three technology replicates within each sample, ***P = 0.0001.

(d) Autophagy flux increased in the STK11IP knock out (KO) HEK293T cells. Bafilomycin A1 treatment was performed at 100 nM for 2 hours.

(e-f) GFP signal decreased in the STK11IP KO HEK293T cells compared to the control cells using the flow cytometry (e) or plate reader (f) analysis. NS: non-starvation. Data are mean ± SEM, n=6 independent biology samples, ***P = 0.0001.

(g) STK11IP deletion leads to enhanced co-localization of LAMP1 (Green color) and LC3 (Red color). Scales bars, 20 μm.

(h) The GFP/RFP ratio increased in the S404A mutation compared to the WT/S404D cells. Data are mean ± SEM, n= 4 independent biological samples. **P = 0.0083, **P=0.0014 respectively.

(i-m) Schematic showing the exercise regimen used in the endurance exercise test (i). The body weight (j), treadmill running distance (k), blood lactate (l), glucose (m) of the WT and STK11IP KO mice were measured. Data are mean ± SEM, n=6 mice each group.

(n) The pS404-STK11IP level decreased upon the endurance treadmill exercise test. Western blot analysis (right panel) and quantification (left panel) analysis of pS404-STK11IP in muscle from the WT mice at rest (-) or after the endurance exercise test (+). n=5 mice each group, Results represent mean ± SEM, *P = 0.0393.

(o-q) Histological sections (H&E stained) (o) form STK11IP WT/KO mice and representative images of the mRFP-GFP-LC3 puncta in the liver (p) and soleus muscle (q) from the WT and STK11IP KO (crossed with mRFP-GFP-LC3 mice) mice after the endurance treadmill exercise. (n = 6 mice per group). Scales bars, 100 μm (o); Scales bars, 10 μm (p: liver), 50 μm (q: Soleus Muscle).

All of the statistical significance was calculated with unpaired two-tailed Student's t tests using the GraphPad Prism software 9. Source data are provided as a Source Data file.

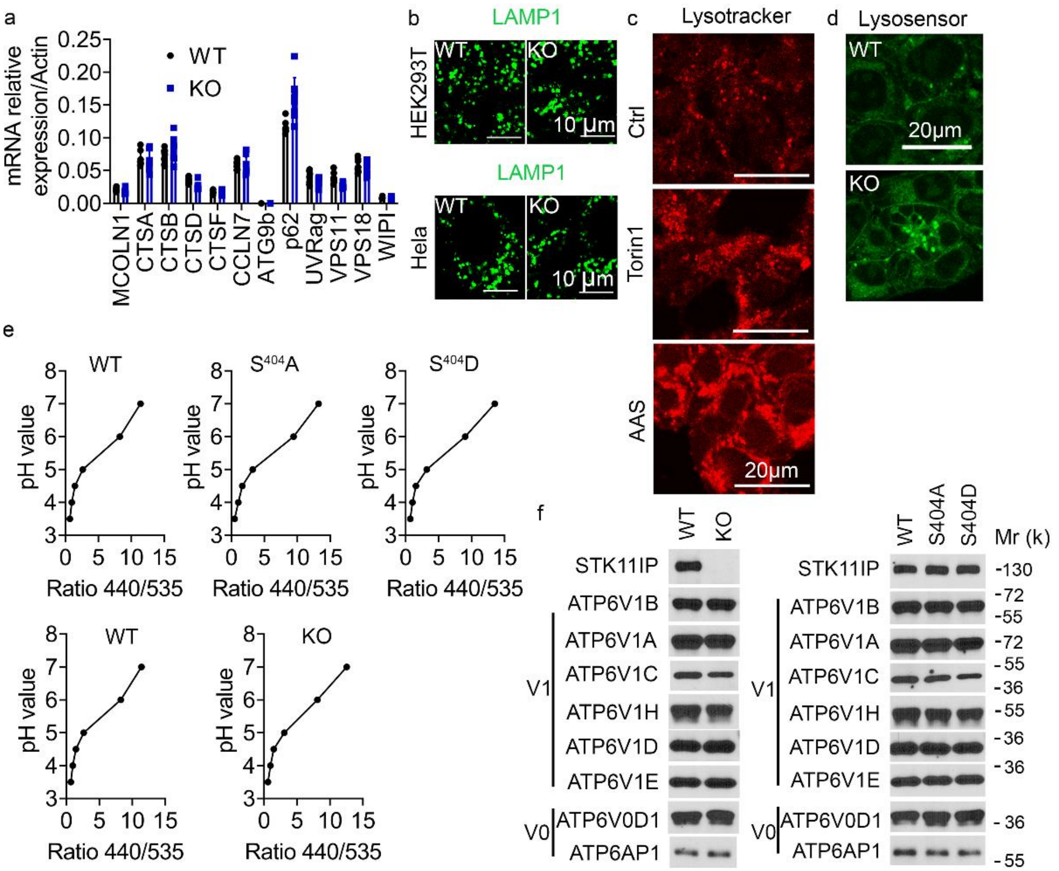

Supplementary Fig. 4. STK11IP Regulates Lysosomal Acidification

(a) STK11IP KO does not affect the expression of lysosomal genes. Data are mean ± SEM, n=3 independent biology samples (with three technology replicates within each sample), Results represent mean ± SEM.

(b) The lysosome morphology showed no difference between wild type and STK11IP knock out (KO) HEK293T or Hela cells. LAMP1 (Green color), scales bars, 10 μm.

(c) Inactivation of mTORC1 by Torin1 (2 μM for 2 hours) or amino acid starvation (2 hours) leads to increased lysosomal acidity. Scales bars, 20 μm.

(d) STK11IP knock out leads to increased lysosomal acidity. Scales bars, 20 μm.

(e) The standard curve for pH measurement using the LysoSensor Yellow/Blue DND-160 dye in the STK11IP WT/KO cells and WT-/S404A-/S404D-STK11IP reconstituted HEK293T cells. (n=4 independent biology samples per point), Results represent mean ± SEM.

(f) Immunoblot analysis of the V-ATPase complex proteins for the in vitro V-ATPase activity assay. The result showed that the same amounts of V-ATPase proteins in the STK11IP WT/KO

experiment and WT-/S404A-/S404D-STK11IP experiment were used for the in vitro V-ATPase activity assay.

Source data are provided as a Source Data file.

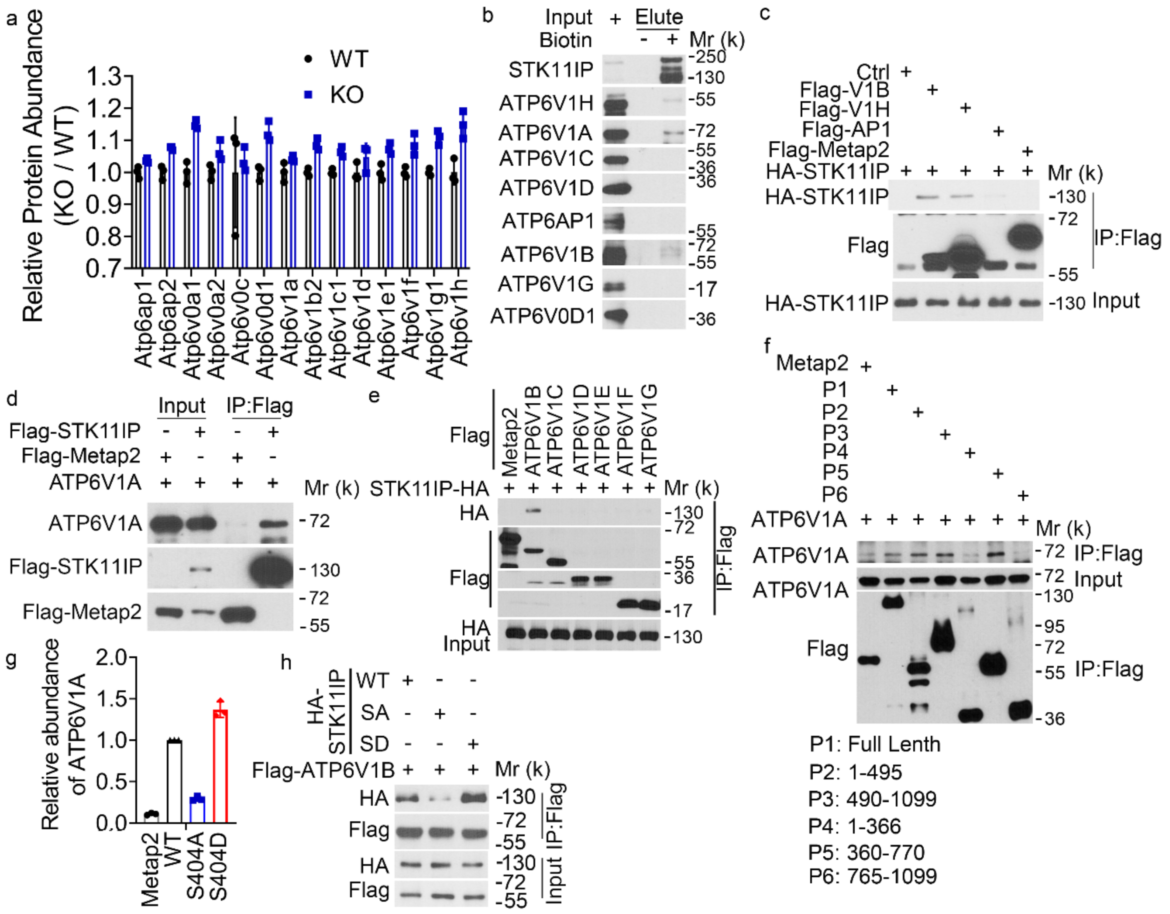

Supplementary Fig. 5. STK11IP Binds to the V-ATPase complex

(a) Expression of the various lysosomal proteins as measured by TMT (tandem mass tag)-based quantitative proteomic experiments. The fold change (KO/WT) of protein abundances of the indicated proteins were extracted and presented. (n=3 independent biology samples each group), Results represent mean ± SEM.

(b) Validation of the interaction between STK11IP and the subunits of V-ATPase complex.

(c) STK11IP interacts with ATP6V1B and ATP6V1H. Flag-Metap2 was used as the negative control.

(d) STK11IP interacts with ATP6V1A. Flag-Metap2 was used as the negative control.

(e) STK11IP did not interact with other subunits of the V1 section of the V-ATPase complex. Flag-Metap2 was used as the negative control, Flag-ATP6V1B was used as the positive control.

(f) Mapping the interaction domain of STK11IP with ATP6V1A. Flag-Metap2 was used as the negative control.

(g) Quantification analysis of Fig. 3m. (n=3 independent biology experiments, Results represent mean ± SEM.)

(h) The STK11IP S404A (SA) mutant interacts more weakly with ATP6V1B, compared to STK11IP WT or the S404D (SD) mutant.

Source data are provided as a Source Data file.

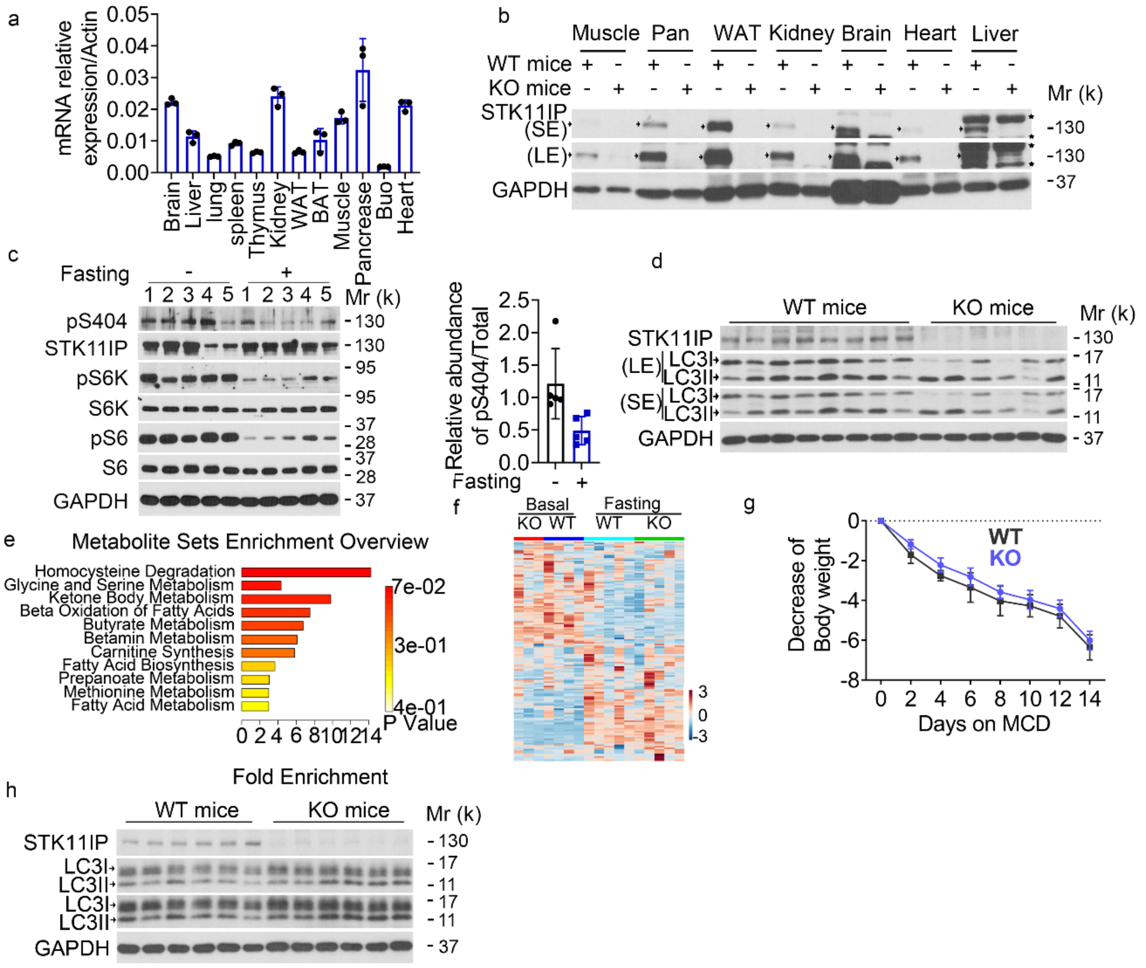

Supplementary Fig. 6. STK11IP Deficiency Protects Mice against Metabolic Disorder

(a-b) Expression levels of STK11IP in various mouse tissues, as measured by RT-qPCR analyses (a) and immunoblot analyses (b). The following tissues were harvested from 2-month-old mice, including the brain, liver, lung, spleen, thymus, kidney, white adipocyte tissue (WAT), brown adipocyte tissue (BAT), muscle, pancreas (Pan), duodenum (Duo) and heart. SE (short exposure); LE (long exposure). (n=3 independent samples), Results represent mean ± SEM. The arrow indicates the STK11IP band, and the asterisk indicates the non-specific band.

(c) The pS404-STK11IP level was decreased upon 36 hours of fasting. Biochemical analysis of pS404-STK11IP in the liver from WT mice at normal (-) or after 36 hours fasting (+) by Western blot detection (Right). Graphs showing quantification of relative ratio pS404/Total-STK11IP at rest and after exercise for 5 mice per group (Left). n=5 independent biology samples, Results represent mean ± SEM.

(d) Immunoblot analysis the LC3II/LC3I ratio in WT and STK11IP KO mice (3-month-old) upon 36 hours of fasting. SE (short exposure); LE (long exposure); (WT, n=8; KO, n=6 mice).

(e) The differentially expressed metabolites were subject to metabolic enrichment analyses using the MetaboAnalyst 5.0 software (WT-basal, n=4; WT-Fasting, n=5; KO-basal, n=3; KO-fasting, n=5).

(f) Pathway enrichment analyses of the serum metabolites that were significantly different between the WT and STK11IP KO mice (3-month-old) after 36 hours fasting (WT-basal, n=4; WT-Fasting, n=5; KO-basal, n=3; KO-fasting, n=5).

(g) Body weight (in grams) of the WT and STK11IP KO mice (2-month-old) that were fed with the MCD diet for 2 weeks (n=8 mice per group). Results represent mean ± SEM.

(h) Immunoblot analysis LC3II/LC3I ratio in the liver from WT and STK11IP KO mice (2-month-old) after 2 weeks MCD diet. SE (short exposure); LE (long exposure); (n = 6 mice per group).

Source data are provided as a Source Data file.

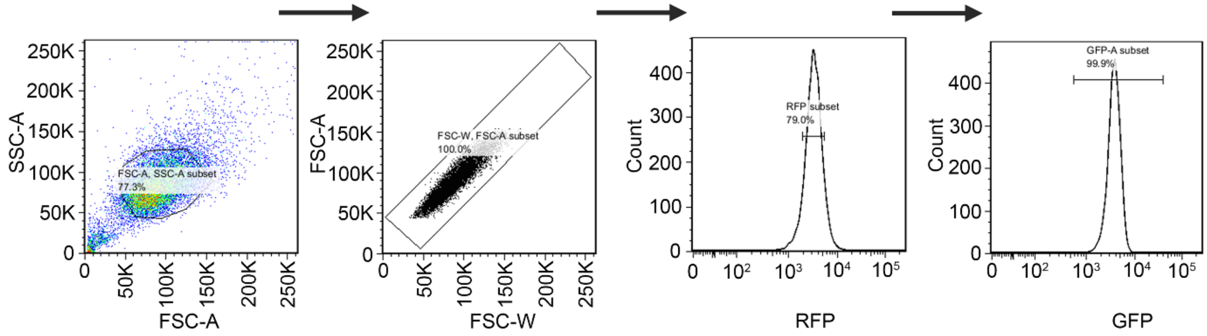

Supplementary Fig. 7. Gating strategies for the FACS analysis.

Using the FSC/SSC gating to remove the debris or attached cells; using the RFP gating to get cells that have similar RFP signals; then compare the GFP signal.

Fig. 1d

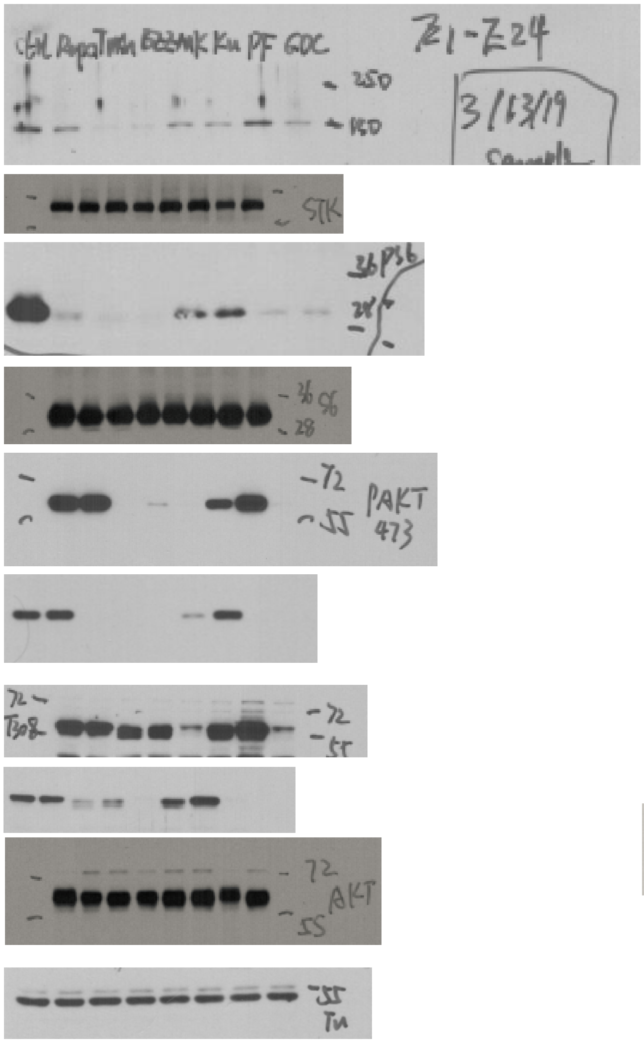

Fig. 1e top panel

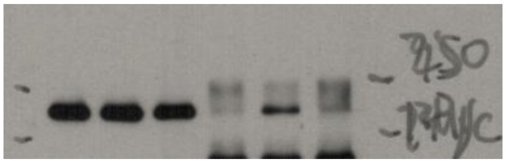

Fig. 2a

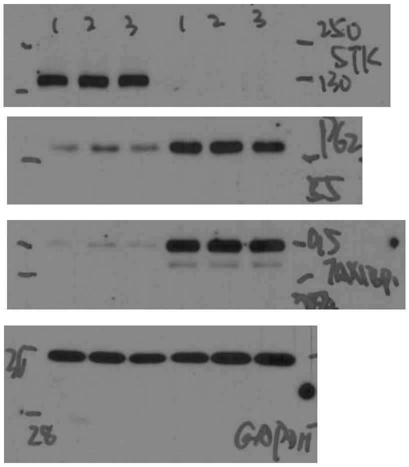

Fig. 2q

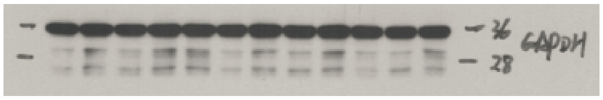

Fig.2s

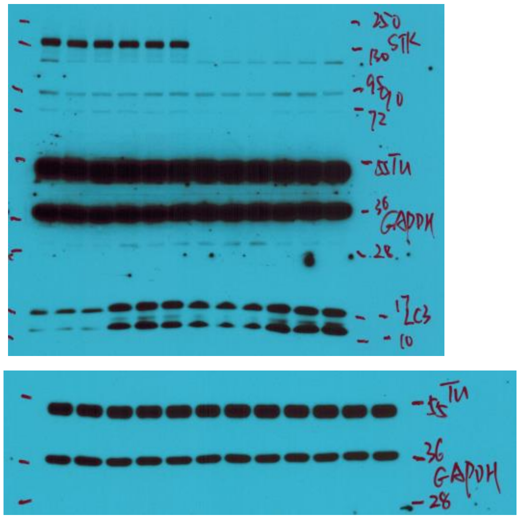

Fig. 2k

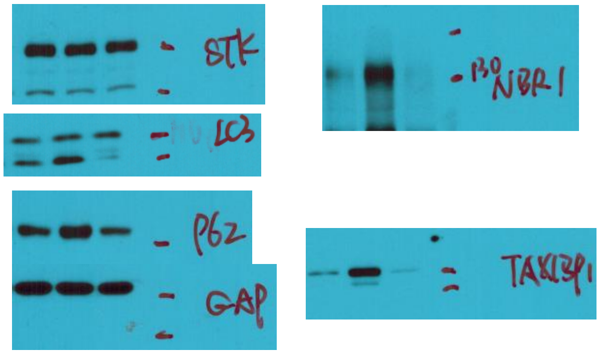

Supplementary Fig. 8. Uncropped immunoblots related to the indicated figures. (Continued on next page)

Fig.3m

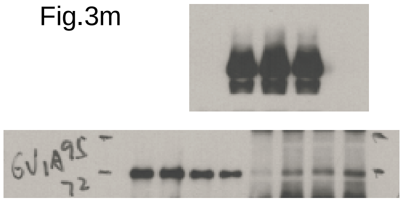

Fig.3n

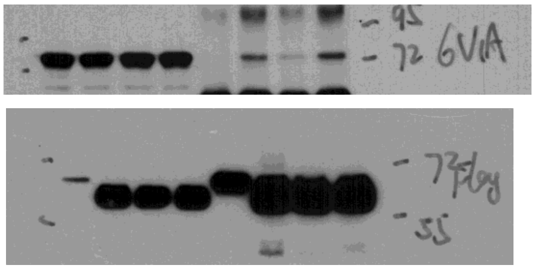

Supplementary Fig. 1a

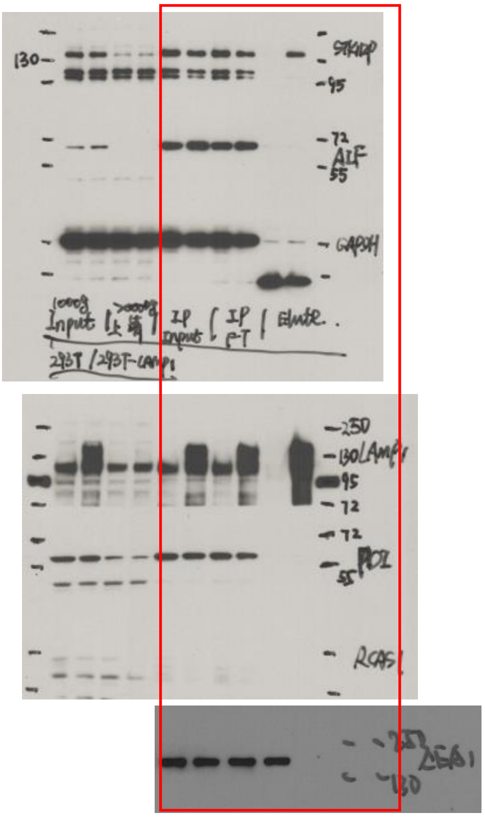

Supplementary Fig. 1b

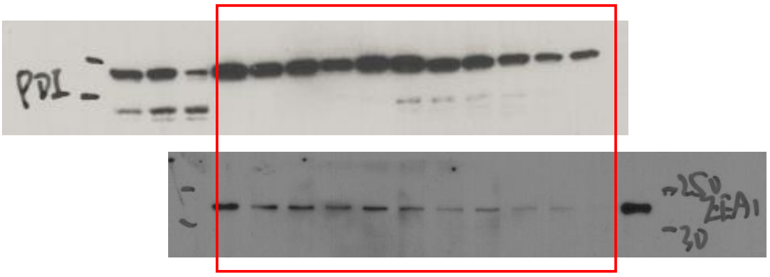

Supplementary Fig. 2a

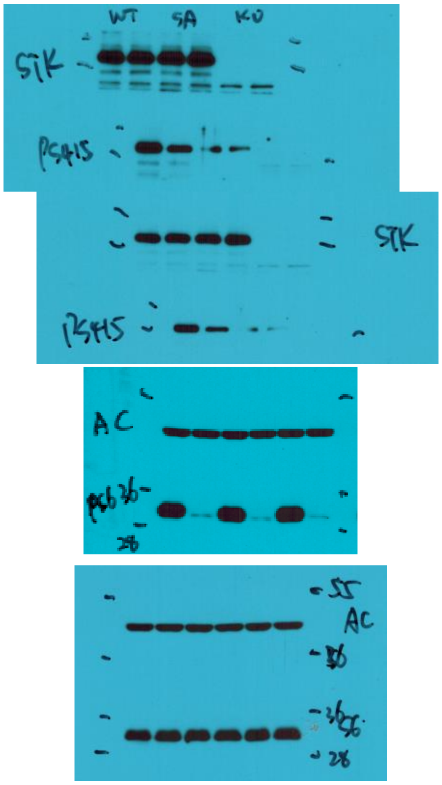

Supplementary Fig. 8. Uncropped immunoblots related to the indicated figures. (Continued from previous)

Supplementary Fig. 2c

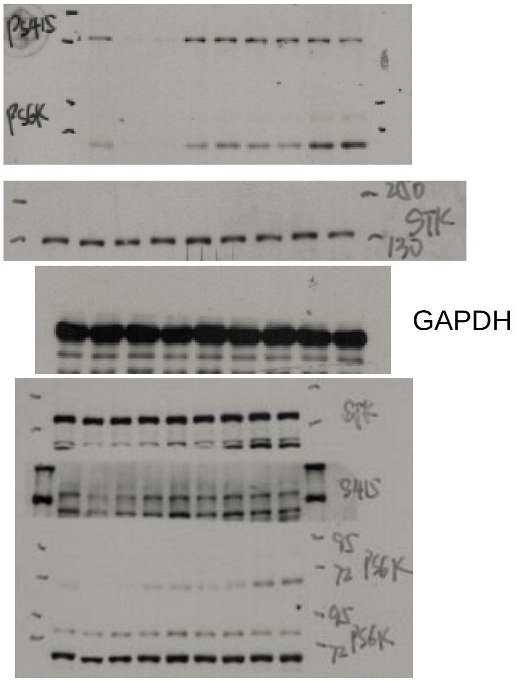

Supplementary Fig. 2d

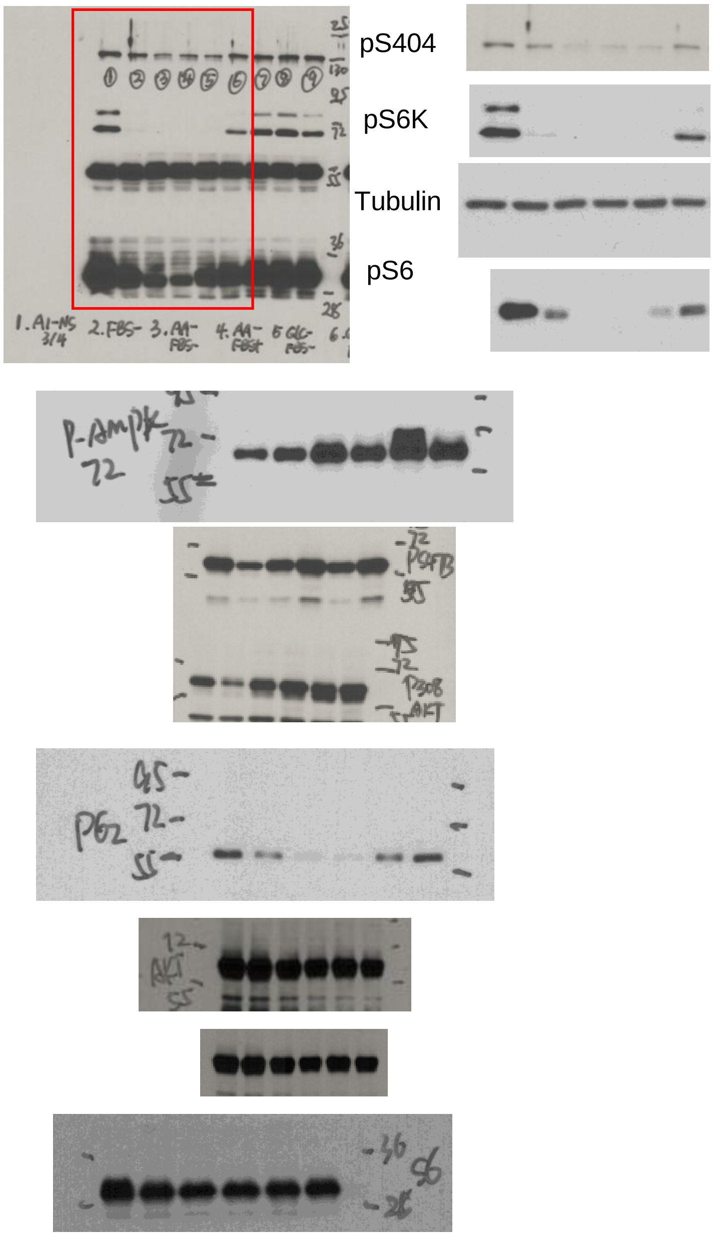

Supplementary Fig. 2e

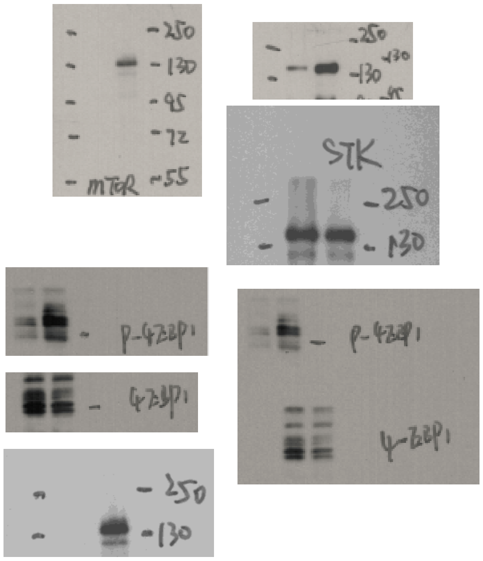

Supplementary Fig. 8. Uncropped immunoblots related to the indicated figures. (Continued from previous)

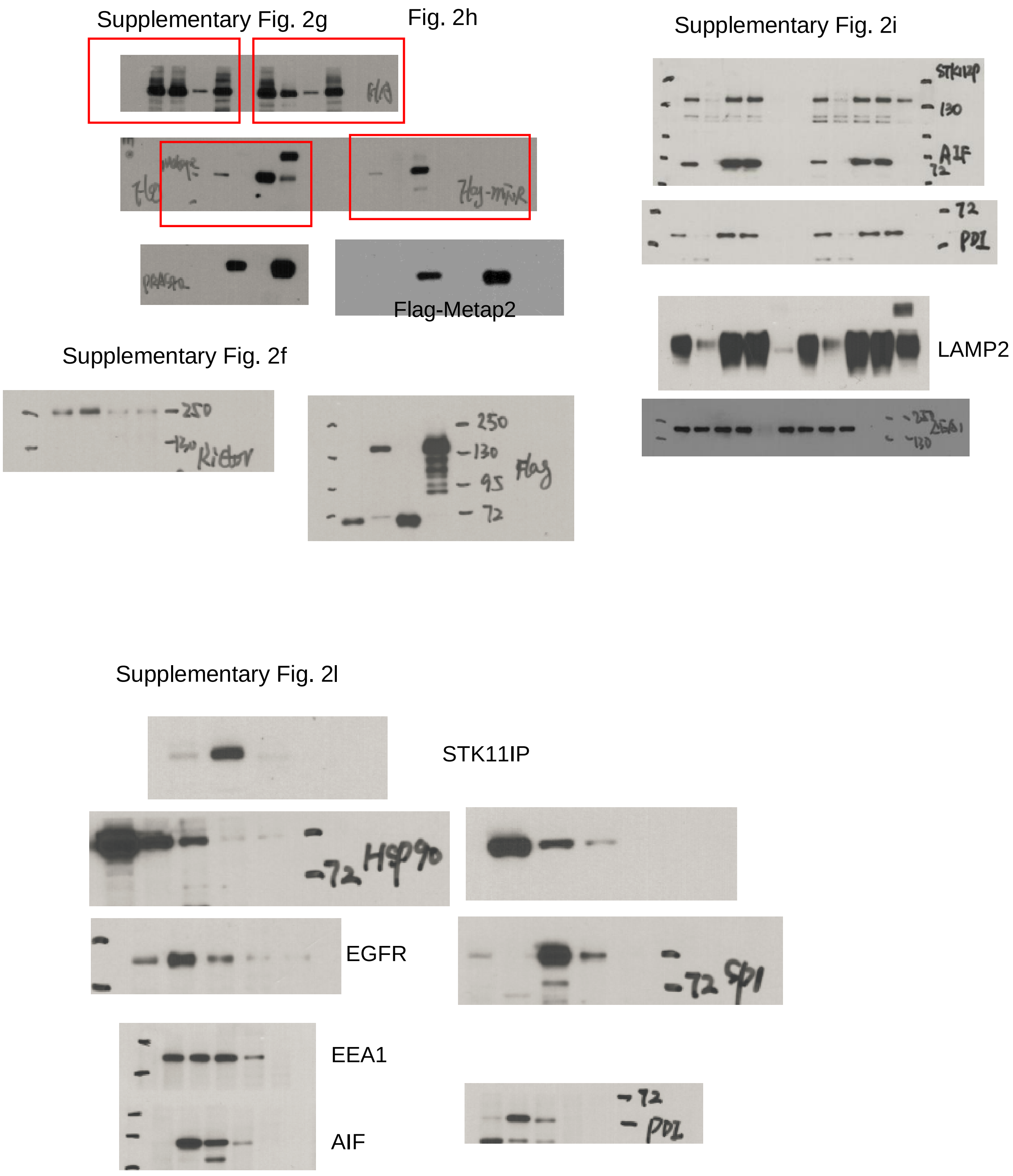

Supplementary Fig. 8. Uncropped immunoblots related to the indicated figures. (Continued from previous)

Supplementary Fig. 3m

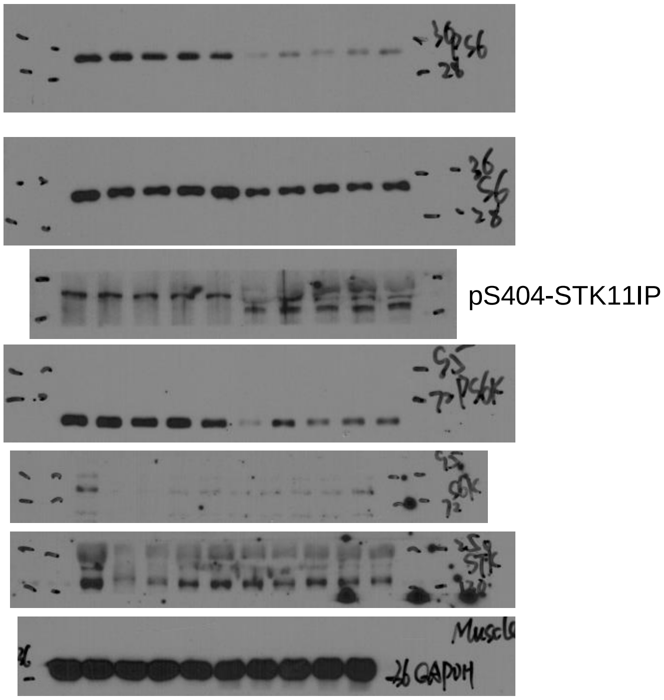

Supplementary Fig. 4f

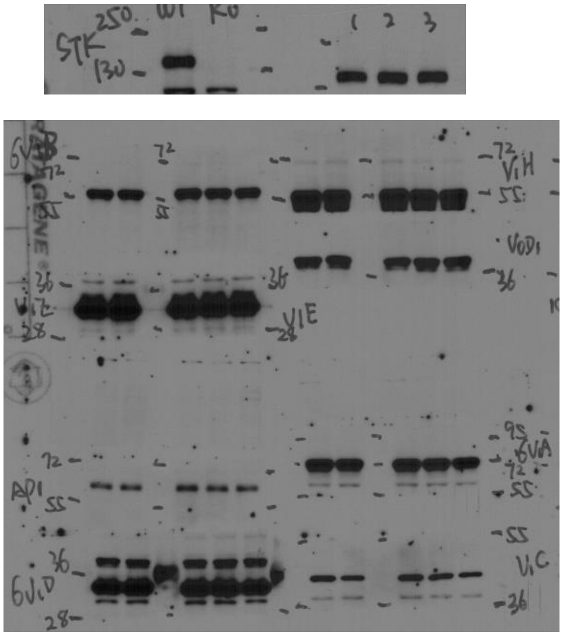

Supplementary Fig. 5b

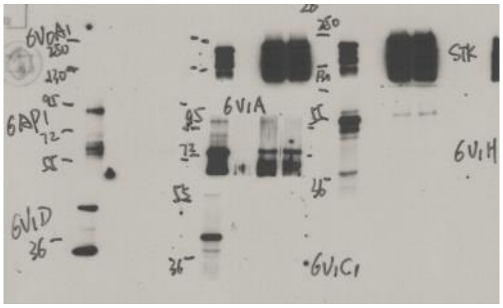

Supplementary Fig. 5c

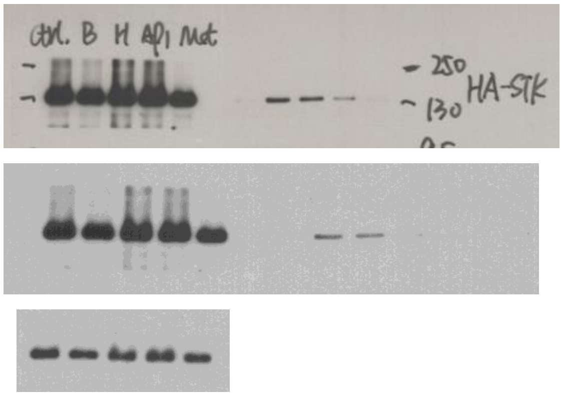

Supplementary Fig. 5d

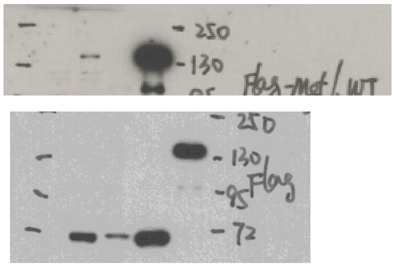

Supplementary Fig. 5f

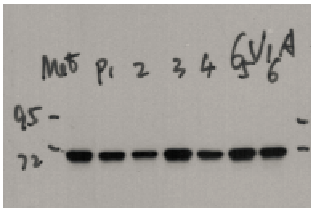

Supplementary Fig. 8. Uncropped immunoblots related to the indicated figures. (Continued from previous)

Supplementary Fig. 5e

HA-Input HA-IP

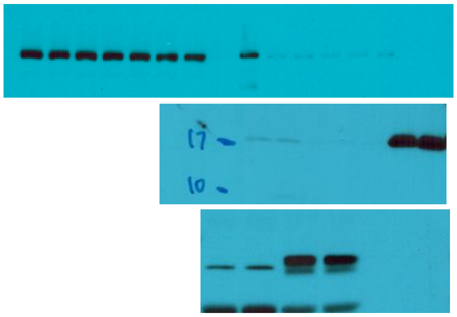

Supplementary Fig. 5h

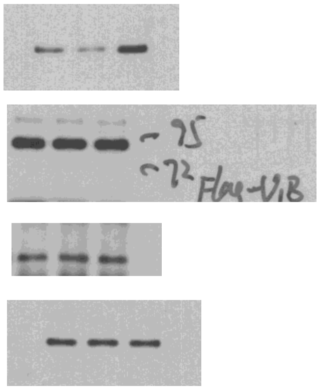

Supplementary Fig. 6c

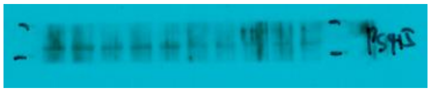

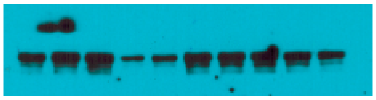

STK11IP

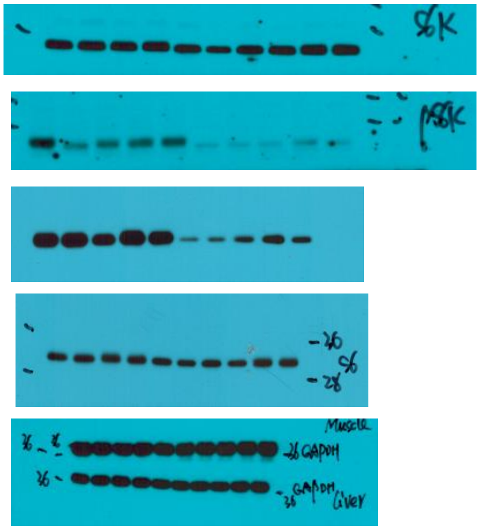

Supplementary Fig. 6d

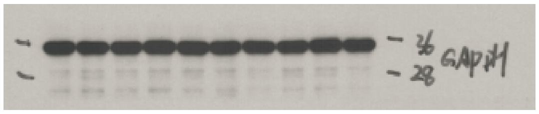

Supplementary Fig. 6h

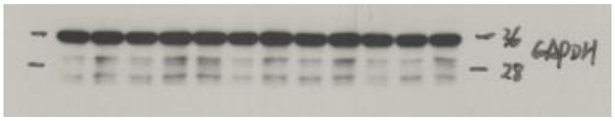

Supplementary Fig. 8. Uncropped immunoblots related to the indicated figures. (Continued from previous)
