# Supplementary material for: Quantitative phosphoproteomic analyses identify STK11IP as a lysosome-specific substrate of mTORC1 that regulates lysosomal acidification
Source: Nat Commun. 2022 Apr 1;13:1760. doi: 10.1038/s41467-022-29461-8 (PMC8976005; doi:10.1038/s41467-022-29461-8)
Supplement: Supplementary file 2 — Description of Additional Supplementary Files [file 41467_2022_29461_MOESM2_ESM.pdf]

Description of Additional Supplementary Files

File name: Supplementary Data 1

Description: Identified Lysosome Proteins using IP-Lysosome-MS Method (related to Supplementary Fig. 1)

File name: Supplementary Data 2

Description: The combined list of phosphopeptides and proteins identified in the Insulin/Akti/Rapa/S6Ki screen. (Related to Fig. 1)

File name: Supplementary Data 3

Description: Expression of the various lysosomal proteins as measured by TMT (tandem mass tag)-based quantitative proteomic experiments. (Related to Supplementary Fig. 5a)

File name: Supplementary Data 4

Description: The proteins identified in the TurboID-STK11IP and Control samples. (Related to Fig. 3k and 3i).

File name: Supplementary Data 5

Description: The metabolomic results identified and quantified by metabolomics experiments using blood from wild type and STK11IP knock out mice after indicated treatment. (Related to Fig. 4i and Supplementary Fig. 6e, 6f)

File name: Supplementary Data 6

Description: The materials including antibodies, plasmid and reagents used in this project.
