# Supplementary material for: Quantitative phosphoproteomic analyses identify STK11IP as a lysosome-specific substrate of mTORC1 that regulates lysosomal acidification
Source: Nat Commun. 2022 Apr 1;13:1760. doi: 10.1038/s41467-022-29461-8 (PMC8976005; doi:10.1038/s41467-022-29461-8)
Supplement: Supplementary file 9 — Reporting Summary [file 41467_2022_29461_MOESM9_ESM.pdf]

nature portfolio

Corresponding author(s): Yonghao Yu

Last updated by author(s): Jul 15, 2021

Reporting Summary

Nature Portfolio wishes to improve the reproducibility of the work that we publish. This form provides structure for consistency and transparency in reporting. For further information on Nature Portfolio policies, see our Editorial Policies and the Editorial Policy Checklist.

Statistics

For all statistical analyses, confirm that the following items are present in the figure legend, table legend, main text, or Methods section.

n/a	Confirmed	
☐	☒	The exact sample size (*n*) for each experimental group/condition, given as a discrete number and unit of measurement
☐	☒	A statement on whether measurements were taken from distinct samples or whether the same sample was measured repeatedly
☐	☒	The statistical test(s) used AND whether they are one- or two-sided<br>*Only common tests should be described solely by name; describe more complex techniques in the Methods section.*
☒	☐	A description of all covariates tested
☐	☒	A description of any assumptions or corrections, such as tests of normality and adjustment for multiple comparisons
☐	☒	A full description of the statistical parameters including central tendency (e.g. means) or other basic estimates (e.g. regression coefficient) AND variation (e.g. standard deviation) or associated estimates of uncertainty (e.g. confidence intervals)
☐	☒	For null hypothesis testing, the test statistic (e.g. *F*, *t*, *r*) with confidence intervals, effect sizes, degrees of freedom and *P* value noted<br>*Give P values as exact values whenever suitable.*
☒	☐	For Bayesian analysis, information on the choice of priors and Markov chain Monte Carlo settings
☒	☐	For hierarchical and complex designs, identification of the appropriate level for tests and full reporting of outcomes
☐	☒	Estimates of effect sizes (e.g. Cohen's *d*, Pearson's *r*), indicating how they were calculated

Our web collection on statistics for biologists contains articles on many of the points above.

Software and code

Policy information about availability of computer code

Data collection	Softwares used for data collection include: Image J 1.50i; CFX Manager 3.0 (Bio-Rad); Microplate Manager 3.05.11; Proteome Discovery (Thermofisher); Sequest algorithm; Flow Cytometry: FACS Aria II SORP (BD); Confocal images were acquired using Zeiss Zen (Zeiss Zen); TEM images were acquired using EM JEOL 1400.
Data analysis	Statistical analysis were performed suing GraphPad Prism 9.1.0 (GraphPad, Inc., La Jolla, CA, USA); Flow Cytometry: FlowJo (v7.6.1); Metabolomic analysis were performed using MetaboAnalysis 5.0 (https://www.metaboanalyst.ca/); GO analysis were performed using DAVID 6.7 (https://david.ncifcrf.gov/home.jsp); Venn Diagram analysis were performed in http://bioinformatics.psb.ugent.be/webtools/Venn/;

For manuscripts utilizing custom algorithms or software that are central to the research but not yet described in published literature, software must be made available to editors and reviewers. We strongly encourage code deposition in a community repository (e.g. GitHub). See the Nature Portfolio guidelines for submitting code & software for further information.

Data

Policy information about availability of data

All manuscripts must include a data availability statement. This statement should provide the following information, where applicable:

- Accession codes, unique identifiers, or web links for publicly available datasets
- A description of any restrictions on data availability
- For clinical datasets or third party data, please ensure that the statement adheres to our policy

All datasets generated and/or analyzed during the study are included in this published article (and its supplementary information files), or avaliable from the corresponding author on reasonale request. The MS data have been deposited in the ProteomeXchange Consortium via the PRIDE partner repository, as described in the data avalibale section.

Field-specific reporting

Please select the one below that is the best fit for your research. If you are not sure, read the appropriate sections before making your selection.

☒ Life sciences ☐ Behavioural & social sciences ☐ Ecological, evolutionary & environmental sciences

For a reference copy of the document with all sections, see nature.com/documents/nr-reporting-summary-flat.pdf

Life sciences study design

All studies must disclose on these points even when the disclosure is negative.

Sample size	Sample size was determined based on the previous studies and literature containing similar procedures.<br>1. The mice endurance exercise test (Exercise-induced BCL2-regulated autophagy is required for muscle glucose homeostasis. Nature 481, 511-515 (2012)); 2. MCD diet induced NASH model using WT and STK11IP-KO mice (Hepatic neuregulin 4 signaling defines an endocrine checkpoint for steatosis-to-NASH progression. J Clin Invest 127, 4449-4461 (2017).).<br>All the sample size information is indicated in the figure legends.<br>For all other experiments, we chose the sample size that is considered to be sufficient to conduct standard statistical tests.
Data exclusions	In Figure 4I and 4J, metabolomic samples in Wild-type basal and STK11IP knockout basal were excluded due to the loss of sample when preparing the samples.<br>For all other experiments, no samples or animals were excluded from analysis.
Replication	All animal experiments and in vitro assays were repeated in at least two independent experiments and the number of repeats was stated in figure legends.
Randomization	All samples were randomly allocated to experimental groups;<br>The mice at the same age and the similar body weight were randomly allocated to experimental groups (such as HFD, MCD and treadmill exercise test).
Blinding	Blinding were performed for the treadmill exercise test, the operator put the mice in different lanes randomly and Investigator observed the exhaustion condition of each mice and harvest the samples, after collecting all the data, the samples were re-identified.<br>The blinding were only applied for the progress of the endurace exercise test, however, for the data analysis as well as other experiments, we compared the phenotype in STK11IP-KO mice versus WT mice, in which no blinding was required because the results are not affected by knowledge of the sample identities.

Reporting for specific materials, systems and methods

We require information from authors about some types of materials, experimental systems and methods used in many studies. Here, indicate whether each material, system or method listed is relevant to your study. If you are not sure if a list item applies to your research, read the appropriate section before selecting a response.

Materials & experimental systems

n/a	Involved in the study	
☐	☒	Antibodies
☐	☒	Eukaryotic cell lines
☒	☐	Palaeontology and archaeology
☐	☒	Animals and other organisms
☒	☐	Human research participants
☒	☐	Clinical data
☒	☐	Dual use research of concern

Methods

n/a	Involved in the study	
☒	☐	ChIP-seq
☐	☒	Flow cytometry
☒	☐	MRI-based neuroimaging

Antibodies

Antibodies used	Anti-GAPDH (Thermo Fisher Scientific, AM4300, Lot# 439919, 1:10000);<br>Anti-LAMP1(Sigma, L1418, Lot# 045M4867V ,1:1000);<br>Anti-LAMP1(Santa Cruz, sc-20011, Lot# F1516, 1:5000);<br>Anti-LAMP2 (Santa Cruz, sc-18822, Lot# D1317, 1:5000);<br>Anti-LC3B (Sigma, L7543, 1:10000);<br>Anti-LC3B (Cell Signaling Technolody, 3868s, Lot#13, 1:200 for IF);<br>Anti-pS404-STK11IP (generated from Thermo Fisher Scientific, using the antigen EPRTLNP(pS)PAGWFV; 1:4000);<br>Anti-STK11IP (Bethyl Lab, A302-464A, 1:2000);<br>Anti-STK11IP (Sigma, HPA036837, Lot# R33597, 1:50 for IF);<br>Anti-pT389-S6K (Cell signaling Technology, 9205, Lot#21 ,1:500);<br>Anti-S6K (Cell Signaling Technology, 9202, Lot#20, 1:1000);

Anti-pS240/244-S6 (Cell Signaling Technology, 5364s, Lot#6,1:10000);
Anti-S6 (Cell Signaling Technology, 2217s, Lot#10, 1:10000);
Anti-pS473-AKT (Cell Signaling Technology, 4060s, Lot#23, 1:1000);
Anti-pT308-AKT (Cell Signaling Technology, 2965s, Lot#18, 1:1000);
Anti-AKT (Cell Signaling Technology, 9272s,Lot#28, 1:1000);
Anti-Tubulin (Cell Signaling Technology, 3873s, Lot#15, 1:10000);
Anti-Flag (Sigma, F7425, 1:5000);
Anti-Flag (Sigma, F1804, Lot#SLBT7654, 1:5000);
Anti-Myc tag (Cell Signaling Technology, 2276s, Lot#4, 1:5000);
Anti-HA (Cell Signaling Technology, 3724s,Lot#9, 1:5000);
Anti-HA (Santa Cruz, sc-7392, 1:5000);
Anti-Tom20 (Santa Cruz, sc-17764, Lot# D2817, 1:1000 for IF);
Anti-ATP6V1A (Santa Cruz, sc-293336, Lot#J3118, 1:1000);
Anti-AIF (Cell Signaling Technology, 5318s, 1:1000);
Anti-PDI (Cell Signaling Technology, 3501s, Lot#2, 1:1000);
Anti-RCAS1 (Cell Signaling Technology, 12290s, Lot#1, 1:1000);
Anti-p62/SQSTM1 (Cell Signaling Technology, 5114s, Lot#5, 1:1000);
Anti-p62/SQSTM1 (Abnova, H00008878-M01, Clone 2C11, 1:10000);
Anti-pT172 (Cell Signaling Technology, 2535s, Lot# 21, 1:1000);
Anti-AMPK (Cell Signaling Technology, 2532s, 1:1000);
Anti-mTORc (Cell Signaling Technology, 2983T, Lot#16, 1:1000);
Anti-4EBP1 (Cell Signaling Technology, 9644s, Lot#12,1:10000);
Anti-pT37/46-4EBP1(Cell signaling Technology, 2855s, Lot#23, 1:10000);
Anti-HSP90 (Cell Signaling Technology, 4877s, 1:1000);
Anti-EGFR (Cell Signaling Technology, 4267s, Lot#2,1:1000);
Anti-SP1 (Cell Signaling Technology, 9839s, Lot#4, 1:1000);
Anti-EEA1 (Cell Signaling Technology, 3288s, 1:1000);
Anti-pS79-ACC (Cell Signaling Technology, 3661s, Lot#4,1:1000);
Anti-pS757-ULK1 (Cell Signaling Technology, 6888s, Lot#3,1:1000);
Anti-ATP6AP1 (Santa Cruz, sc-81886, Lot#J1117, 1:1000);
Anti-ATP6V1H (Santa Cruz, sc-166227, Lot#H0817, 1:1000);
Anti-ATP6V1C (Santa Cruz, sc-271077, Lot#H1017, 1:1000);
Anti-ATP6V1D (Santa Cruz, sc-166218, Lot#F1814, 1:1000);
Anti-ATP6V1B (Santa Cruz, sc-55544, Lot#J1817, 1:1000);
Anti-ATP6V1G (Santa Cruz, sc-25333, Lot#H1417, 1:1000);
Anti-ATP6V0D1 (Santa Cruz, sc-393322, Lot#C0218,1:1000);
Anti-TAX1BP1 (Cell Signalling Technology, 5105s, 1:1000);
Anti-EEA1 (Cell Signalling Technology, 48453s, 1:1000);
Anti-NBR1 (Santa Cruz, sc-130380, 1:1000);
Donkey anti Rabbit IgG (GE healthcare, NA9340, 1:10000);
Goat anti Mouse IgG (Millipore, AP181P, 1:10000);
Donkey anti-Mouse IgG (H+L) Cross-Adsorbed Secondary Antibody, Alexa Fluor 488(Thermos Fisher, A21202, 1;1000);
Donkey anti-Rabbit IgG (H+L) Cross-Adsorbed Secondary Antibody, Alexa Fluor 594 (Thermos Fisher, A21207, 1:1000).

Validation

All antibodies from commercial company are well-validated by manufacturer and widely used in the scientific research for western blotting or Immuno-staining.
Anti-p-S404-STK11IP antibody was generated by Thermo Fisher Scientific, using the antigen EPRTLNP(pS)PAGWFV from human) and validated through the S404A mutation and mTORC1 related inhibitor (Torin1) treatment (These results were shown in Supplementary Fig. 2a and 2b).
Anti-GAPDH (Thermo Fisher Scientific, AM4300, https://www.thermofisher.com/antibody/product/GAPDH-Antibody-clone-6C5-Monoclonal/AM4300)
Anti-LAMP1(Sigma, L1418,https://www.sigmaaldrich.com/catalog/product/sigma/l1418?lang=en&region=US)
Anti-LAMP1(Santa Cruz, sc-20011, https://www.scbt.com/p/lamp-1-antibody-h4a3)
Anti-LAMP2 (Santa Cruz, sc-18822, https://www.scbt.com/p/lamp-2-antibody-h4b4?requestFrom=search)
Anti-LC3B (Sigma, L7543, https://www.sigmaaldrich.com/catalog/product/sigma/l7543?lang=en&region=US)
Anti-LC3B (Cell Signaling Technolody, 3868s, https://www.cellsignal.com/products/primary-antibodies/lc3b-d11-xp-rabbit-mab/3868?site-search-type=Products&N=4294956287&Ntt=lc3b+&fromPage=plp)
Anti-STK11IP (Bethyl Lab, A302-464A,https://www.bethyl.com/product/A302-464A/STK11IP+Antibody)
Anti-STK11IP (Sigma, HPA036837, https://www.sigmaaldrich.com/catalog/product/sigma/hpa036837?lang=en&region=US)
Anti-pT389-S6K (Cell signaling Technology, 9205s, https://www.cellsignal.com/products/primary-antibodies/phospho-p70-s6-kinase-thr389-antibody/9205)
Anti-S6K (Cell Signaling Technology, 9202s, https://www.cellsignal.com/products/primary-antibodies/p70-s6-kinase-antibody/9202);
Anti-pS240/244-S6 (Cell Signaling Technology, 5364s, https://www.cellsignal.com/products/primary-antibodies/phospho-s6-ribosomal-protein-ser240-244-d68f8-xp-rabbit-mab/5364);
Anti-S6 (Cell Signaling Technology, 2217s, https://www.cellsignal.com/products/primary-antibodies/s6-ribosomal-protein-5g10-rabbit-mab/2217?site-search-type=Products&N=4294956287&Ntt=s6+ribosomal+protein&fromPage=plp);
Anti-pS473-AKT (Cell Signaling Technology, 4060s, https://www.cellsignal.com/products/primary-antibodies/phospho-akt-ser473-d9e-xp-rabbit-mab/4060);
Anti-pT308-AKT (Cell Signaling Technology, 2965s, https://www.cellsignal.com/products/primary-antibodies/phospho-akt-thr308-c31e5e-rabbit-mab/2965);
Anti-AKT (Cell Signaling Technology, 9272s,https://www.cellsignal.com/products/primary-antibodies/akt-antibody/9272?site-search-type=Products&N=4294956287&Ntt=%099272&fromPage=plp&_requestid=5927792);
Anti-Tubulin (Cell Signaling Technology, 3873s, https://www.cellsignal.com/products/primary-antibodies/a-tubulin-dm1a-mouse-mab/3873?site-search-type=Products&N=4294956287&Ntt=3873s&fromPage=plp&_requestid=5928008);
Anti-Flag (Sigma, F7425, https://www.sigmaaldrich.com/catalog/product/sigma/f7425?lang=en&region=US);
Anti-Flag (Sigma, A8592, https://www.sigmaaldrich.com/catalog/product/sigma/a8592?lang=en&region=US);

Anti-Myc tag (Cell Signaling Technology, 2276s, https://www.cellsignal.com/products/primary-antibodies/myc-tag-9b11-mouse-mab/2276);
Anti-HA (Cell Signaling Technology, 3724s, https://www.cellsignal.com/products/primary-antibodies/ha-tag-c29f4-rabbit-mab/3724);
Anti-HA (Santa Cruz, sc-7392, https://www.scbt.com/p/ha-probe-antibody-f-7);
Anti-Tom20 (Santa Cruz, sc-17764, https://www.scbt.com/p/tom20-antibody-f-10?requestFrom=search);
Anti-ATP6V1A (Santa Cruz, sc-293336, https://www.scbt.com/p/v-atpase-alpha1-antibody-4f5?requestFrom=search);
Anti-AIF (Cell Signaling Technology, 5318s, https://www.cellsignal.com/products/primary-antibodies/aif-d39d2-xp-rabbit-mab/5318);
Anti-PDI (Cell Signaling Technology, 3501s, https://www.cellsignal.com/products/primary-antibodies/pdi-c81h6-rabbit-mab/3501);
Anti-RCAS1 (Cell Signaling Technology, 12290s, https://www.cellsignal.com/products/primary-antibodies/rcas1-d2b6n-xp-rabbit-mab/12290?_=1616545385905&Ntt=12290&tahead=true);
Anti-p62/SQSTM1 (Cell Signaling Technology, 5114s, https://www.cellsignal.com/products/primary-antibodies/sqstm1-p62-antibody/5114);
Anti-p62/SQSTM1 (Abnova, H00008878-M01, http://www.abnova.com/products/products_detail.asp?catalog_id=H00008878-M01);
Anti-pT172 (Cell Signaling Technology, 2535s, https://www.cellsignal.com/products/primary-antibodies/phospho-ampka-thr172-40h9-rabbit-mab/2535);
Anti-AMPK (Cell Signaling Technology, 2532s, https://www.cellsignal.com/products/primary-antibodies/ampka-antibody/2532?N=4294956287&Ntt=AMPK&fromPage=plp);
Anti-mTORc (Cell Signaling Technology, 2983T, https://www.cellsignal.com/products/primary-antibodies/mtor-7c10-rabbit-mab/2983?_=1616546528751&Ntt=2983&tahead=true);
Anti-4EBP1 (Cell Signaling Technology, 9644s, https://www.cellsignal.com/products/primary-antibodies/4e-bp1-53h11-rabbit-mab/9644?site-search-type=Products&N=4294956287&Ntt=4ebp1&fromPage=plp);
Anti-pT37/46-4EBP1(Cell signaling Technology, 2855s, https://www.cellsignal.com/products/primary-antibodies/phospho-4e-bp1-thr37-46-236b4-rabbit-mab/2855);
Anti-HSP90 (Cell Signaling Technology, 4877s, https://www.cellsignal.com/products/primary-antibodies/hsp90-c45g5-rabbit-mab/4877?site-search-type=Products&N=4294956287&Ntt=hsp90&fromPage=plp);
Anti-EGFR (Cell Signaling Technology, 4267s, https://www.cellsignal.com/products/primary-antibodies/egf-receptor-d38b1-xp-rabbit-mab/4267);
Anti-SP1 (Cell Signaling Technology, 9839s, https://www.cellsignal.com/products/primary-antibodies/sp1-d4c3-rabbit-mab/9389);
Anti-EEA1 (Cell Signaling Technology, 3288s, https://www.cellsignal.com/products/primary-antibodies/eea1-c45b10-rabbit-mab/3288);
Anti-pS79-ACC (Cell Signaling Technology, 3661s, https://www.cellsignal.com/products/primary-antibodies/phospho-acetyl-coa-carboxylase-ser79-antibody/3661);
Anti-pS757-ULK1 (Cell Signaling Technology, 6888s, https://www.cellsignal.com/products/primary-antibodies/phospho-ulk1-ser757-antibody/6888);
Anti-ATP6AP1 (Santa Cruz, sc-81886, https://www.scbt.com/p/atp6ap1-antibody-85-1);
Anti-ATP6V1H (Santa Cruz, sc-166227, https://www.scbt.com/p/v-atpase-h-antibody-c-8?requestFrom=search);
Anti-ATP6V1C (Santa Cruz, sc-271077, https://www.scbt.com/p/v-atpase-c1-antibody-g-5?requestFrom=search);
Anti-ATP6V1D (Santa Cruz, sc-166218, https://www.scbt.com/p/v-atpase-d-antibody-d-4);
Anti-ATP6V1B (Santa Cruz, sc-55544, https://www.scbt.com/p/v-atpase-b1-2-antibody-f-6?requestFrom=search);
Anti-ATP6V1G (Santa Cruz, sc-25333, https://www.scbt.com/p/v-atpase-g1-antibody-d-5?requestFrom=search);
Anti-ATP6V0D1 (Santa Cruz, sc-393322, https://www.scbt.com/p/v-atpase-d1-antibody-d-4?requestFrom=search);
Anti-EEA1 (Cell Signalling technology, 48453s, https://www.cellsignal.com/products/primary-antibodies/eea1-e9q6g-mouse-mab/48453);
Anti-TAX1BP1 (Cell Signalling technology, 5105s, https://www.cellsignal.com/products/primary-antibodies/tax1bp1-d1d5-rabbit-mab/5105);
Anti-NBR1 (Santa Cruz, sc-130380, https://www.scbt.com/p/nbr1-antibody-4br);
Goat anti Mouse IgG (Millipore, AP181P, https://www.emdmillipore.com/US/en/product/Goat-Anti-Mouse-IgG-Antibody-HRP-conjugate-Species-Adsorbed,MM_NF-AP181P);
Donkey anti-Mouse IgG (H+L) Cross-Adsorbed Secondary Antibody, Alexa Fluor 488(Thermos Fisher, A21202, https://www.thermofisher.com/antibody/product/Donkey-anti-Mouse-IgG-H-L-Highly-Cross-Adsorbed-Secondary-Antibody-Polyclonal/A-21202);
Donkey anti-Rabbit IgG (H+L) Cross-Adsorbed Secondary Antibody, Alexa Fluor 594 (Thermos Fisher, A21207, https://www.thermofisher.com/antibody/product/Donkey-anti-Rabbit-IgG-H-L-Highly-Cross-Adsorbed-Secondary-Antibody-Polyclonal/A-21207).

Eukaryotic cell lines

Policy information about cell lines

Cell line source(s)	HEK293T cells (ATCC, Ca# CRL-3216) and Hela cells (ATCC, Ca# CCL-2) were purchased from ATCC; Primary MEF cells were isolated from the mice at E12.5.
Authentication	No further authentication of the cell lines was performed before use.
Mycoplasma contamination	The cells were not tested for Mycoplasma contamination.
Commonly misidentified lines (See ICLAC register)	No commonly misidentified cell lines was used in this study.

Animals and other organisms

Policy information about studies involving animals; ARRIVE guidelines recommended for reporting animal research

Laboratory animals	Mice used in this study were stated in the method and figure legends.

Laboratory animals	Wild-type male C57BL/6NJ mice (005304), STK11IP knockout mice (028999) and RFP-GFP-LC3 transgenic mice (027139) were purchased from the JAX lab. All the mice used in this study are maintained on a C57BL/6NJ background. STK11IP-/- mice were back-crossed to 6th generation. In all the reported data, male mice (from 8 to 12 weeks old) were used.
Wild animals	No wild mice were used in this study. All experiment animals were housed in a barrier specific pathogen free animal facility with 12 hr light/12 hr dark cycle with free access to water and food.
Field-collected samples	No field-collected samples were involved.
Ethics oversight	All performed procedures on mice have been approved by Institutional Animal Care and Use Committee (IACUC) of UT Southwestern Medical Center (APN#2017-102133).

Note that full information on the approval of the study protocol must also be provided in the manuscript.

Flow Cytometry

Plots

Confirm that:

☒ The axis labels state the marker and fluorochrome used (e.g. CD4-FITC).

☒ The axis scales are clearly visible. Include numbers along axes only for bottom left plot of group (a 'group' is an analysis of identical markers).

☒ All plots are contour plots with outliers or pseudocolor plots.

☒ A numerical value for number of cells or percentage (with statistics) is provided.

Methodology

Sample preparation	Cells were typically grown in 6 well, washed, trypsinized, collected on ice, washed and resuspended in FACS buffer (DPBS, 2% FBS, and 0.2 mM EDTA).
Instrument	BD FACS Aria II SORP (analysis); BD FACS Aria Fusion (Sorting)
Software	BD FACSDiva, FlowJo (V7.6.1)
Cell population abundance	10,000 cells were gating for GFP-LC3-RFP signal analysis.
Gating strategy	Using the FSC/SSC gating to remove the debris or attached cells; using the RFP gating to get cells that have similar RFP signals; then compare the GFP signal. The gating strategied has been incorpearted in Supplementary Fig. 7.

☒ Tick this box to confirm that a figure exemplifying the gating strategy is provided in the Supplementary Information.
